# Supplementary material for: Regional excess mortality during the 2020 COVID-19 pandemic in five European countries
Source: Nat Commun. 2022 Jan 25;13:482. doi: 10.1038/s41467-022-28157-3 (PMC8789777; doi:10.1038/s41467-022-28157-3)

Online Supplement of "Regional excess mortality during the 2020 COVID-19 pandemic in five European countries"

Garyfallos Konstantinoudis[1,*], Michela Cameletti[2], Virgilio Gómez-Rubio[3], Inmaculada León Gómez[4,5], Monica Pirani[1], Gianluca Baio[6], Amparo Larrauri[4,5], Julien Riou[7], Matthias Egger[7, 8], Paolo Vineis[1], and Marta Blangiardo[1]

[1]MRC Centre for Environment and Health, Department of Epidemiology and Biostatistics, School of Public Health, Imperial College London, London, UK

[2]Department of Economics, University of Bergamo, Bergamo, Italy

[3]Departamento de Matemáticas, Escuela Técnica Superior de Ingenieros Industriales, Universidad de Castilla-La Mancha, Albacete, Spain

[4]National Centre of Epidemiology (CNE), Institute of Health Carlos III, Madrid, Spain

[5]Consortium for Biomedical Research in Epidemiology and Public Health (CIBERESP), Institute of Health Carlos III, Madrid, Spain

[6]Department of Statistical Sciences, University College London, UK

[7]Institute of Social and Preventive Medicine, University of Bern, Bern, Switzerland

[8]Population Health Sciences, Bristol Medical School, University of Bristol, Bristol, UK

[*]Corresponding author, email: `g.konstantinoudis@imperial.ac.uk`

List of Supplementary Tables

1 Cross-validation results: correlation between predicted and observed number of deaths, and 95% coverage probability across the different countries and age and sex groups. 6
2 Median relative excess deaths and 95% credible intervals in 2020 per NUTS2 regions in England. 7
3 Median relative excess deaths and 95% credible intervals in 2020 per NUTS2 regions in Greece. . 8
4 Median relative excess deaths and 95% credible intervals in 2020 per NUTS2 regions in Italy. . . 9
5 Median relative excess deaths and 95% credible intervals in 2020 per NUTS2 regions in Spain. . . 10
6 Median relative excess deaths and 95% credible intervals in 2020 per NUTS2 regions in Switzerland. 11
7 Frequency of population by age and sex groups in England, Greece, Italy, Spain and Switzerland in 2019. 12
8 Data sources for deaths and population across the different countries. 13

List of Supplementary Figures

1 NUTS2 (black) and NUTS3 (grey) region borders in England, Greece, Italy, Spain and Switzerland. 14
2 Relative mortality risk of the effect of temperature during 2015 and 2020 by age and sex groups in England. 15
3 Relative mortality risk of the effect of temperature during 2015 and 2020 by age and sex groups in Greece. 16
4 Relative mortality risk of the effect of temperature during 2015 and 2020 by age and sex groups in Italy. 17
5 Relative mortality risk of the effect of temperature during 2015 and 2020 by age and sex groups in Spain. 18
6 Relative mortality risk of the effect of temperature during 2015 and 2020 by age and sex groups in Switzerland. 19
7 Weekly trends for all-cause total number of deaths by gender and age in England. The blue curve represents the posterior mean predicted from the 2015–2019 model, while the shaded blue ribbon describes the posterior 95% interval. The black line shows the observed number of deaths for 2020. 20
8 Weekly trends for all-cause total number of deaths by gender and age in Greece. The blue curve represents the posterior mean predicted from the 2015–2019 model, while the shaded blue ribbon describes the posterior 95% interval. The black line shows the observed number of deaths for 2020. 21

9 Weekly trends for all-cause total number of deaths by gender and age in Italy. The blue curve represents the posterior mean predicted from the 2015–2019 model, while the shaded blue ribbon describes the posterior 95% interval. The black line shows the observed number of deaths for 2020. 22
10 Weekly trends for all-cause total number of deaths by gender and age in Spain. The blue curve represents the posterior mean predicted from the 2015–2019 model, while the shaded blue ribbon describes the posterior 95% interval. The black line shows the observed number of deaths for 2020. 23
11 Weekly trends for all-cause total number of deaths by gender and age in Switzerland. The blue curve represents the posterior mean predicted from the 2015–2019 model, while the shaded blue ribbon describes the posterior 95% interval. The black line shows the observed number of deaths for 2020. 24
12 Relative excess mortality during 2020 by NUTS3 regions in England stratified by age and sex. . . 25
13 Posterior probability that the relative excess mortality is larger than zero during 2020 by NUTS3 regions in England stratified by age and sex. 26
14 Relative excess mortality during 2020 by NUTS3 regions in Greece stratified by age and sex. . . 27
15 Posterior probability that the relative excess mortality is larger than zero during 2020 by NUTS3 regions in Greece stratified by age and sex. 28
16 Relative excess mortality during 2020 by NUTS3 regions in Italy stratified by age and sex. 29
17 Posterior probability that the relative excess mortality is larger than zero during 2020 by NUTS3 regions in Italy stratified by age and sex. 30
18 Relative excess mortality during 2020 by NUTS3 regions in Spain stratified by age and sex. . . . 31
19 Posterior probability that the relative excess mortality is larger than zero during 2020 by NUTS3 regions in Spain stratified by age and sex. 32
20 Relative excess mortality during 2020 by NUTS3 regions in Switzerland stratified by age and sex. 33
21 Posterior probability that the relative excess mortality is larger than zero during 2020 by NUTS3 regions in Switzerland stratified by age and sex. 34
22 Relative excess mortality during January-June 2020 by NUTS3 regions in England stratified by age and sex. 35
23 Relative excess mortality during July-December 2020 by NUTS3 regions in England stratified by age and sex. 36
24 Posterior probability that the relative excess mortality is larger than zero during January-June 2020 by NUTS3 regions in England stratified by age and sex. 37

25 Posterior probability that the relative excess mortality is larger than zero during July-December 2020 by NUTS3 regions in England stratified by age and sex. 38
26 Relative excess mortality during January-June 2020 by NUTS3 regions in Greece stratified by age and sex. 39
27 Relative excess mortality during July-December 2020 by NUTS3 regions in Greece stratified by age and sex. 40
28 Posterior probability that the relative excess mortality is larger than zero during January-June 2020 by NUTS3 regions in Greece stratified by age and sex. 41
29 Posterior probability that the relative excess mortality is larger than zero during July-December 2020 by NUTS3 regions in Greece stratified by age and sex. 42
30 Relative excess mortality during January-June 2020 by NUTS3 regions in Italy stratified by age and sex. 43
31 Relative excess mortality during July-December 2020 by NUTS3 regions in Italy stratified by age and sex. 44
32 Posterior probability that the relative excess mortality is larger than zero during January-June 2020 by NUTS3 regions in Italy stratified by age and sex. 45
33 Posterior probability that the relative excess mortality is larger than zero during July-December 2020 by NUTS3 regions in Italy stratified by age and sex. 46
34 Relative excess mortality during January-June 2020 by NUTS3 regions in Spain stratified by age and sex. 47
35 Relative excess mortality during July-December 2020 by NUTS3 regions in Spain stratified by age and sex. 48
36 Posterior probability that the relative excess mortality is larger than zero during January-June 2020 by NUTS3 regions in Spain stratified by age and sex. 49
37 Posterior probability that the relative excess mortality is larger than zero during July-December 2020 by NUTS3 regions in Spain stratified by age and sex. 50
38 Relative excess mortality during January-June 2020 by NUTS3 regions in Switzerland stratified by age and sex. 51
39 Relative excess mortality during July-December 2020 by NUTS3 regions in Switzerland stratified by age and sex. 52

40 Posterior probability that the relative excess mortality is larger than zero during January-June 2020 by NUTS3 regions in Switzerland stratified by age and sex. 53
41 Posterior probability that the relative excess mortality is larger than zero during July-December 2020 by NUTS3 regions in Switzerland stratified by age and sex. 54

Supplementary Table 1: Cross-validation results: correlation between predicted and observed number of deaths, and 95% coverage probability across the different countries and age and sex groups.

Males										
	England		Greece		Italy		Spain		Switzerland	
Age group	Correlation	Coverage	Correlation	Coverage	Correlation	Coverage	Correlation	Coverage	Correlation	Coverage
< 40	0.23 (0.21, 0.24)	0.94	0.56 (0.57, 0.62)	0.92	0.51 (0.50, 0.52)	0.94	0.69 (0.68, 0.70)	0.93	0.41 (0.38, 0.44)	0.93
40-59	0.45 (0.44, 0.46)	0.95	0.87 (0.86, 0.87)	0.94	0.83 (0.83, 0.84)	0.95	0.90 (0.90, 0.91)	0.94	0.69 (0.67, 0.71)	0.94
60-69	0.55 (0.54, 0.55)	0.95	0.90 (0.89, 0.90)	0.95	0.87 (0.85, 0.88)	0.95	0.92 (0.92, 0.93)	0.94	0.74 (0.72, 0.76)	0.94
70-79	0.70 (0.69, 0.71)	0.95	0.93 (0.92, 0.93)	0.95	0.93 (0.91, 0.93)	0.95	0.95 (0.94, 0.95)	0.93	0.84 (0.83, 0.85)	0.95
$geq80$	0.83 (0.82, 0.84)	0.94	0.96 (0.95, 0.96)	0.95	0.95 (0.94, 0.95)	0.93	0.97 (0.97, 0.97)	0.91	0.92 (0.91, 0.93)	0.95
Females										
$40 <$	0.15 (0.14, 0.16)	0.93	0.43 (0.40, 0.46)	0.88	0.38 (0.37, 0.40)	0.92	0.58 (0.56, 0.59)	0.92	0.28 (0.26, 0.31)	0.89
40-59	0.35 (0.34, 0.36)	0.95	0.81 (0.79, 0.81)	0.93	0.78 (0.77, 0.79)	0.95	0.86 (0.85, 0.86)	0.94	0.57 (0.55, 0.59)	0.93
60-69	0.46 (0.45, 0.47)	0.95	0.86 (0.85, 0.86)	0.94	0.83 (0.82, 0.83)	0.95	0.88 (0.87, 0.88)	0.95	0.64 (0.63, 0.66)	0.94
70-79	0.65 (0.63, 0.65)	0.95	0.92 (0.91, 0.92)	0.95	0.91 (0.90, 0.92)	0.95	0.92 (0.91, 0.93)	0.94	0.81 (0.80, 0.82)	0.94
$geq80$	0.87 (0.82, 0.84)	0.94	0.97 (0.96, 0.97)	0.94	0.96 (0.95, 0.96)	0.92	0.97 (0.97, 0.98)	0.90	0.94 (0.93, 0.95)	0.94

Supplementary Table 2: Median relative excess deaths and 95% credible intervals in 2020 per NUTS2 regions in England.

Region	Males	Females
Outer London - West and North West	0.21 (0.15, 0.28)	0.12 (0.05, 0.28)
Outer London - East and North East	0.20 (0.14, 0.26)	0.15 (0.07, 0.26)
West Midlands	0.19 (0.13, 0.25)	0.14 (0.06, 0.25)
Inner London - East	0.19 (0.13, 0.26)	0.15 (0.08, 0.26)
Outer London - South	0.16 (0.10, 0.24)	0.08 (-0.00, 0.24)
South Yorkshire	0.16 (0.10, 0.22)	0.12 (0.05, 0.22)
Lancashire	0.15 (0.09, 0.21)	0.08 (0.01, 0.21)
Greater Manchester	0.14 (0.09, 0.20)	0.09 (0.02, 0.20)
West Yorkshire	0.14 (0.08, 0.20)	0.08 (0.01, 0.20)
Kent	0.14 (0.07, 0.20)	0.08 (0.01, 0.20)
Merseyside	0.13 (0.07, 0.19)	0.10 (0.02, 0.19)
Leicestershire, Rutland and Northamptonshire	0.13 (0.07, 0.19)	0.07 (-0.01, 0.19)
Bedfordshire and Hertfordshire	0.12 (0.06, 0.18)	0.08 (0.00, 0.18)
Shropshire and Staffordshire	0.11 (0.06, 0.18)	0.07 (0.00, 0.18)
Tees Valley and Durham	0.11 (0.05, 0.17)	0.09 (0.00, 0.17)
Essex	0.11 (0.05, 0.17)	0.03 (-0.04, 0.17)
Cumbria	0.10 (0.03, 0.17)	0.06 (-0.02, 0.17)
Herefordshire, Worcestershire and Warwickshire	0.10 (0.04, 0.16)	0.07 (-0.00, 0.16)
East Yorkshire and Northern Lincolnshire	0.09 (0.03, 0.16)	0.04 (-0.03, 0.16)
Inner London - West	0.09 (0.03, 0.16)	0.02 (-0.06, 0.16)
Derbyshire and Nottinghamshire	0.09 (0.04, 0.15)	0.04 (-0.04, 0.15)
Lincolnshire	0.09 (0.03, 0.16)	0.06 (-0.02, 0.16)
Northumberland and Tyne and Wear	0.08 (0.03, 0.14)	0.05 (-0.02, 0.14)
Berkshire, Buckinghamshire and Oxfordshire	0.08 (0.02, 0.14)	0.06 (-0.02, 0.14)
Surrey, East and West Sussex	0.07 (0.01, 0.12)	0.05 (-0.03, 0.12)
Cheshire	0.07 (0.01, 0.13)	0.06 (-0.02, 0.13)
Gloucestershire, Wiltshire and Bath/Bristol area	0.06 (0.01, 0.12)	0.04 (-0.03, 0.12)
Hampshire and Isle of Wight	0.04 (-0.01, 0.10)	0.02 (-0.05, 0.10)
East Anglia	0.04 (-0.01, 0.09)	0.03 (-0.05, 0.09)
North Yorkshire	0.04 (-0.02, 0.10)	0.03 (-0.05, 0.10)
Dorset and Somerset	0.02 (-0.03, 0.08)	-0.00 (-0.08, 0.08)
Cornwall and Isles of Scilly	-0.02 (-0.08, 0.04)	-0.05 (-0.11, 0.04)
Devon	-0.03 (-0.08, 0.03)	-0.05 (-0.12, 0.03)
England	0.11 (0.05, 0.16)	0.06 (-0.01, 0.16)

Supplementary Table 3: Median relative excess deaths and 95% credible intervals in 2020 per NUTS2 regions in Greece.

Region	Males	Females
Eastern Macedonia and Thrace	0.17 (0.09, 0.25)	0.14 (0.06, 0.25)
Western Macedonia	0.16 (0.08, 0.27)	0.19 (0.08, 0.27)
Central Macedonia	0.15 (0.08, 0.24)	0.13 (0.05, 0.24)
Thessaly	0.08 (0.01, 0.17)	0.09 (0.01, 0.17)
Western Greece	0.05 (-0.02, 0.12)	0.01 (-0.07, 0.12)
Ionian Islands	0.04 (-0.04, 0.13)	0.01 (-0.08, 0.13)
Attica	0.02 (-0.04, 0.09)	0.02 (-0.05, 0.09)
Central Greece	0.02 (-0.05, 0.10)	-0.00 (-0.08, 0.10)
Epirus	-0.00 (-0.08, 0.09)	0.01 (-0.07, 0.09)
Peloponnese	-0.00 (-0.07, 0.08)	0.05 (-0.04, 0.08)
Crete	-0.01 (-0.08, 0.06)	0.02 (-0.06, 0.06)
North Aegean	-0.01 (-0.10, 0.08)	0.06 (-0.04, 0.08)
South Aegean	-0.03 (-0.10, 0.05)	0.02 (-0.06, 0.05)
Greece	0.06 (-0.01, 0.13)	0.06 (-0.02, 0.13)

Supplementary Table 4: Median relative excess deaths and 95% credible intervals in 2020 per NUTS2 regions in Italy.

Region	Males	Females
Lombardia	0.29 (0.20, 0.38)	0.25 (0.17, 0.38)
Piemonte	0.18 (0.10, 0.27)	0.17 (0.08, 0.27)
Trentino-Alto Adige	0.16 (0.08, 0.25)	0.20 (0.11, 0.25)
Valle d'Aosta	0.15 (0.05, 0.28)	0.23 (0.10, 0.28)
Liguria	0.15 (0.06, 0.23)	0.12 (0.04, 0.23)
Emilia-Romagna	0.13 (0.04, 0.21)	0.12 (0.04, 0.21)
Veneto	0.10 (0.02, 0.17)	0.11 (0.02, 0.17)
Marche	0.07 (-0.01, 0.15)	0.08 (0.01, 0.15)
Puglia	0.07 (-0.00, 0.14)	0.05 (-0.02, 0.14)
Friuli Venezia Giulia	0.06 (-0.02, 0.13)	0.08 (-0.00, 0.13)
Sardegna	0.05 (-0.02, 0.12)	0.06 (-0.02, 0.12)
Abruzzo	0.04 (-0.04, 0.11)	0.01 (-0.06, 0.11)
Toscana	0.03 (-0.04, 0.11)	0.04 (-0.03, 0.11)
Campania	0.03 (-0.03, 0.10)	-0.00 (-0.07, 0.10)
Sicilia	0.02 (-0.05, 0.09)	0.02 (-0.05, 0.09)
Basilicata	0.02 (-0.05, 0.10)	0.03 (-0.06, 0.10)
Umbria	0.01 (-0.06, 0.09)	0.01 (-0.07, 0.09)
Calabria	0.01 (-0.06, 0.08)	0.00 (-0.07, 0.08)
Lazio	0.00 (-0.07, 0.06)	-0.02 (-0.09, 0.06)
Molise	-0.00 (-0.08, 0.08)	0.06 (-0.03, 0.08)
Italy	0.10 (0.03, 0.18)	0.09 (0.02, 0.18)

Supplementary Table 5: Median relative excess deaths and 95% credible intervals in 2020 per NUTS2 regions in Spain.

Region	Males	Females
Madrid	0.33 (0.27, 0.39)	0.28 (0.20, 0.35)
Castile-la Mancha	0.32 (0.26, 0.38)	0.32 (0.23, 0.40)
Castile-leon	0.28 (0.22, 0.34)	0.28 (0.20, 0.36)
La Rioja	0.18 (0.11, 0.28)	0.16 (0.06, 0.26)
Catalonia	0.15 (0.09, 0.20)	0.18 (0.10, 0.25)
Aragon	0.15 (0.09, 0.21)	0.20 (0.12, 0.28)
Extremadura	0.12 (0.06, 0.18)	0.15 (0.07, 0.23)
Ceuta	0.11 (-0.02, 0.30)	0.31 (0.14, 0.54)
Navarre	0.10 (0.04, 0.17)	0.15 (0.07, 0.23)
Principality Of Asturias	0.08 (0.03, 0.13)	0.10 (0.03, 0.16)
Basque Community	0.07 (0.02, 0.12)	0.09 (0.02, 0.16)
Region Of Murcia	0.04 (-0.01, 0.10)	0.02 (-0.04, 0.09)
Cantabria	0.04 (-0.02, 0.10)	0.06 (-0.01, 0.14)
Andalusia	0.03 (-0.01, 0.08)	0.04 (-0.02, 0.10)
Valencian Community	0.03 (-0.02, 0.07)	0.02 (-0.04, 0.08)
Melilla	-0.01 (-0.13, 0.16)	0.13 (-0.01, 0.30)
Galicia	-0.02 (-0.06, 0.03)	0.01 (-0.06, 0.07)
Canary Islands	-0.08 (-0.12, -0.03)	-0.05 (-0.10, 0.01)
Balearic Islands	-0.08 (-0.13, -0.04)	-0.07 (-0.13, -0.01)
Spain	0.12 (0.06, 0.19)	0.12 (0.06, 0.19)

Supplementary Table 6: Median relative excess deaths and 95% credible intervals in 2020 per NUTS2 regions in Switzerland.

Region	Males	Females
Ticino	0.20 (0.11, 0.32)	0.18 (0.09, 0.32)
Lake Geneva region	0.16 (0.08, 0.25)	0.14 (0.07, 0.25)
Eastern Switzerland	0.08 (0.00, 0.16)	0.07 (0.01, 0.16)
Espace Mittelland	0.08 (0.00, 0.16)	0.05 (-0.01, 0.16)
Northwestern Switzerland	0.06 (-0.01, 0.15)	0.04 (-0.02, 0.15)
Central Switzerland	0.05 (-0.02, 0.13)	0.01 (-0.05, 0.13)
Zurich	0.02 (-0.06, 0.10)	0.05 (-0.01, 0.10)
Switzerland	0.08 (0.01, 0.16)	0.07 (0.01, 0.16)

Supplementary Table 7: Frequency of population by age and sex groups in England, Greece, Italy, Spain and Switzerland in 2019.

Age	Sex	England	Greece	Italy	Spain	Switzerland
40<	female	0.25	0.23	0.19	0.21	0.23
40<	male	0.25	0.23	0.20	0.22	0.24
40-59	female	0.13	0.15	0.16	0.16	0.14
40-59	male	0.13	0.14	0.15	0.16	0.15
60-69	female	. 0.05	0.06	0.06	0.06	0.06
60-69	male	0.05	0.06	0.06	0.05	0.05
70-79	female	0.04	0.05	0.05	0.04	0.04
70-79	male	0.04	0.04	0.05	0.04	0.04
80+	female	. 0.03	0.02	0.05	0.04	0.03
80+	male	0.02	0.02	0.03	0.02	0.02

Supplementary Table 8: Data sources for deaths and population across the different countries.

country	Deaths	Population	Time point
England	Small Area Health Statistics Unit (SAHSU)	Office for National Statistics (ONS)	30-06
Greece	Hellenic Statistical Authority (ELSTAT)	ELSTAT	01-01
Italy	Italian National Institute of Statistics (ISTAT)	ISTAT	01-01
Spain	National Statistics Institute (INE)	INE	01-01
Switzerland	Federal Statistical Office (BFS)	BFS	31-12

Supplementary Figure 1: NUTS2 (black) and NUTS3 (grey) region borders in England, Greece, Italy, Spain and Switzerland.

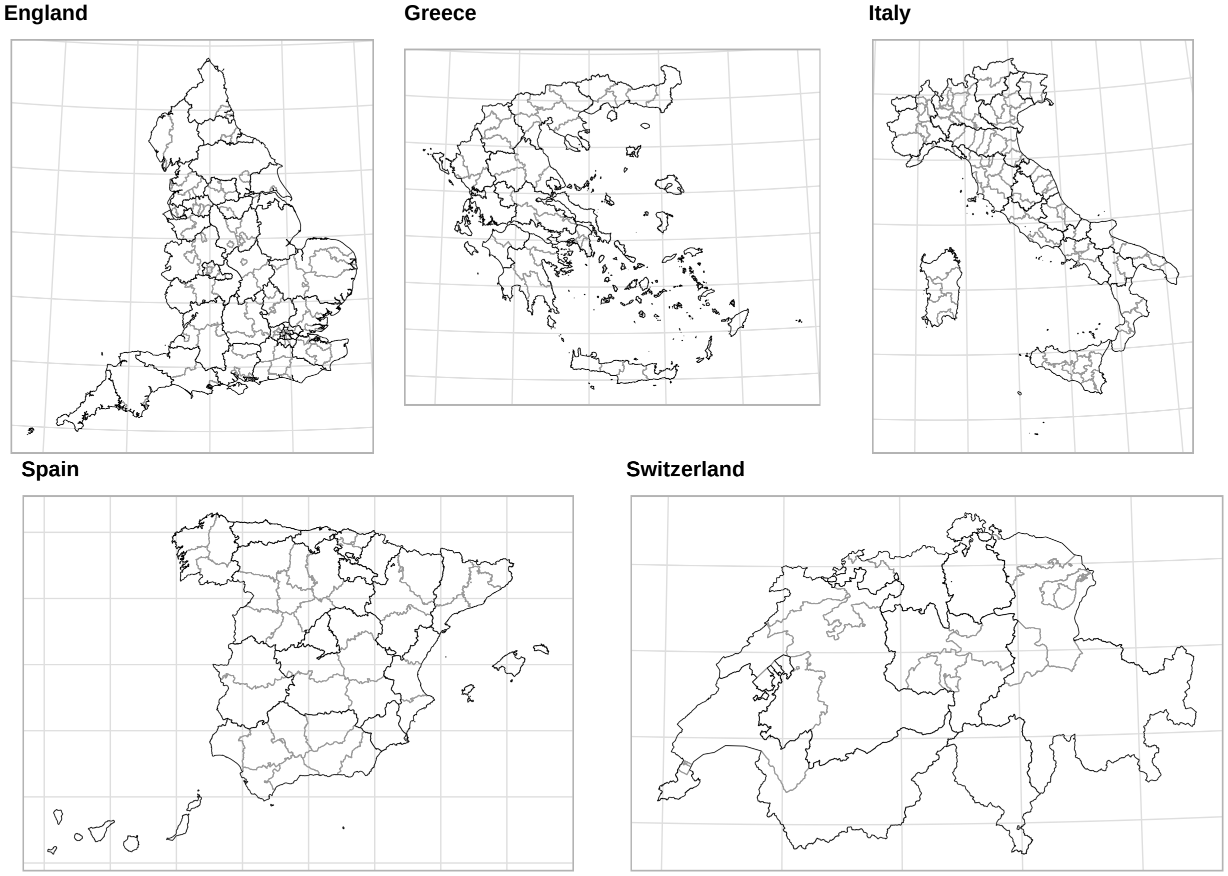

Supplementary Figure 2: Relative mortality risk of the effect of temperature during 2015 and 2020 by age and sex groups in England.

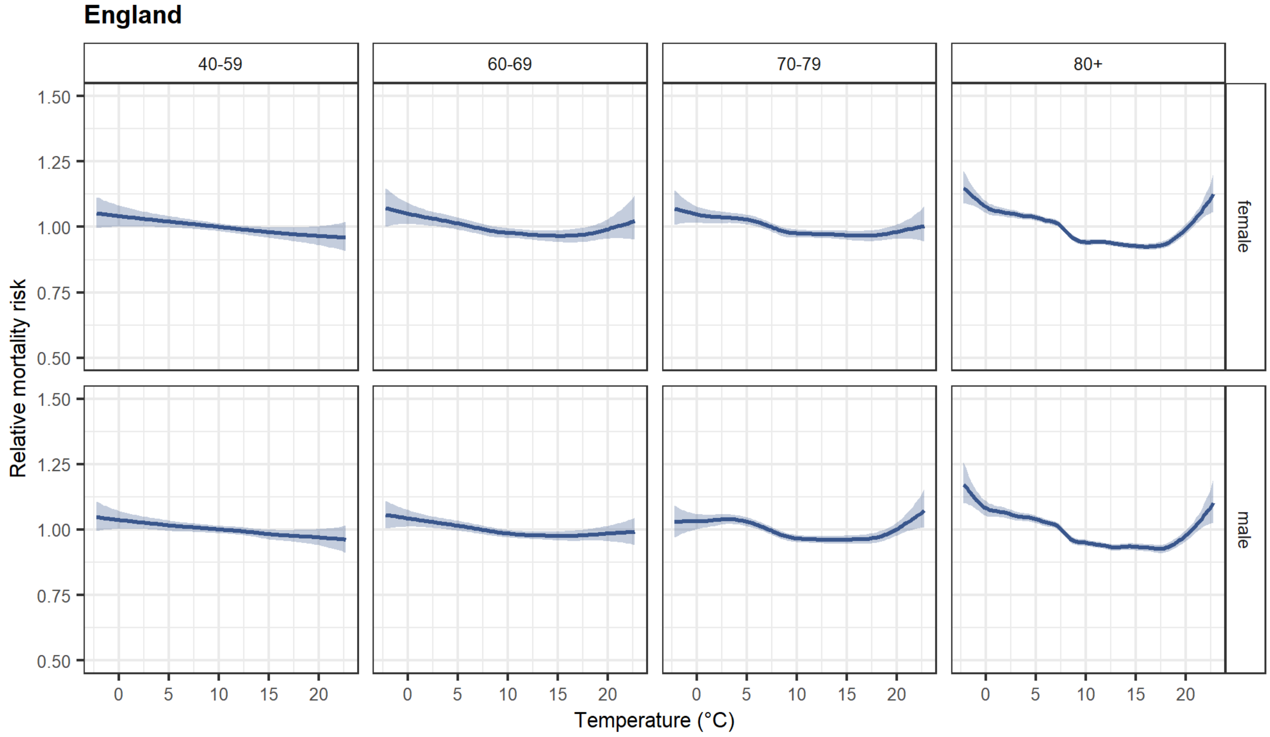

112 Data are presented as median relative mortality risk (the blue line) and 95% Credible Intervals (shaded blue
113 areas).

Supplementary Figure 3: Relative mortality risk of the effect of temperature during 2015 and 2020 by age and sex groups in Greece.

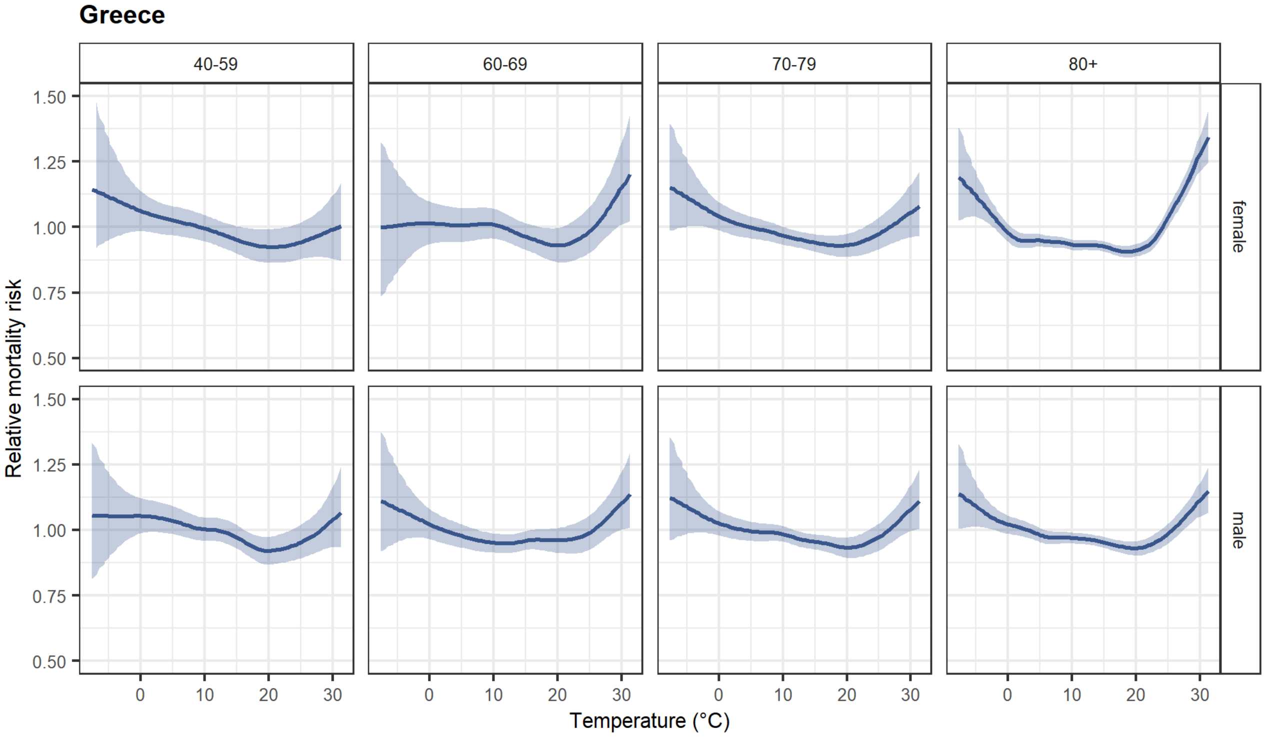

Data are presented as median relative mortality risk (the blue line) and 95% Credible Intervals (shaded blue areas).

Supplementary Figure 4: Relative mortality risk of the effect of temperature during 2015 and 2020 by age and sex groups in Italy.

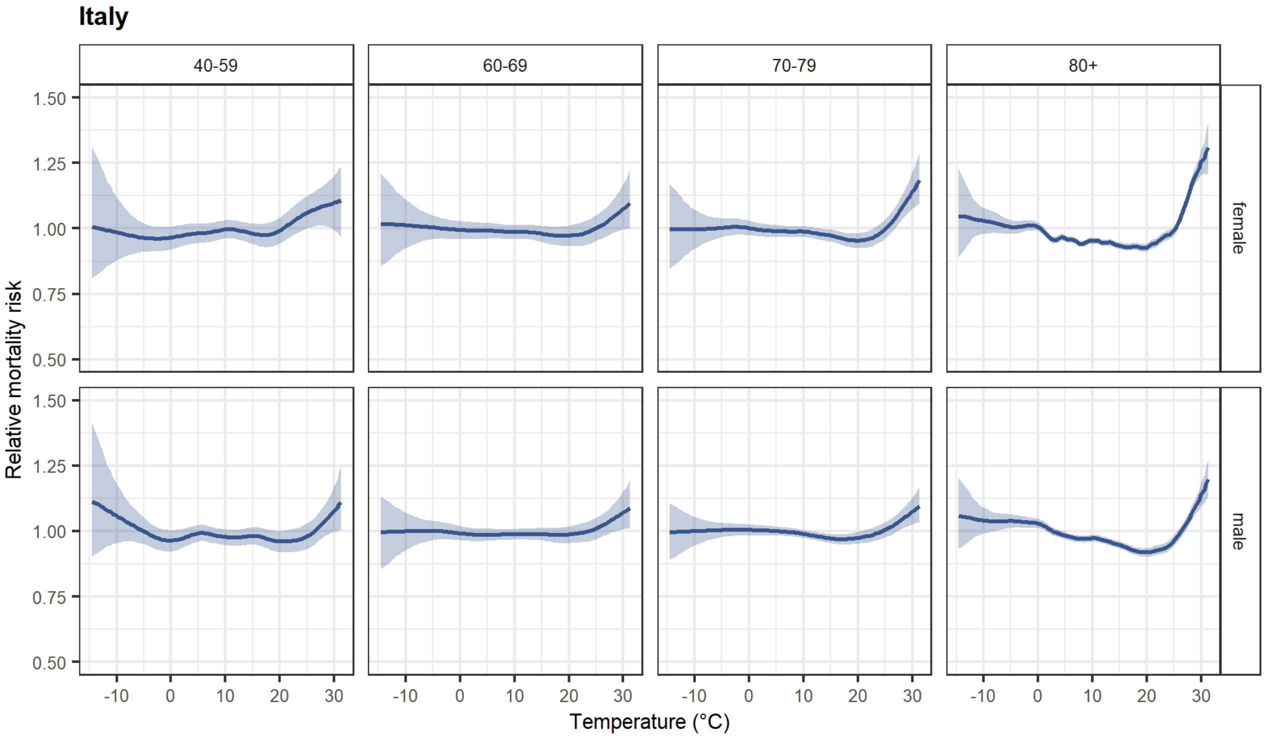

Data are presented as median relative mortality risk (the blue line) and 95% Credible Intervals (shaded blue areas).

Supplementary Figure 5: Relative mortality risk of the effect of temperature during 2015 and 2020 by age and sex groups in Spain.

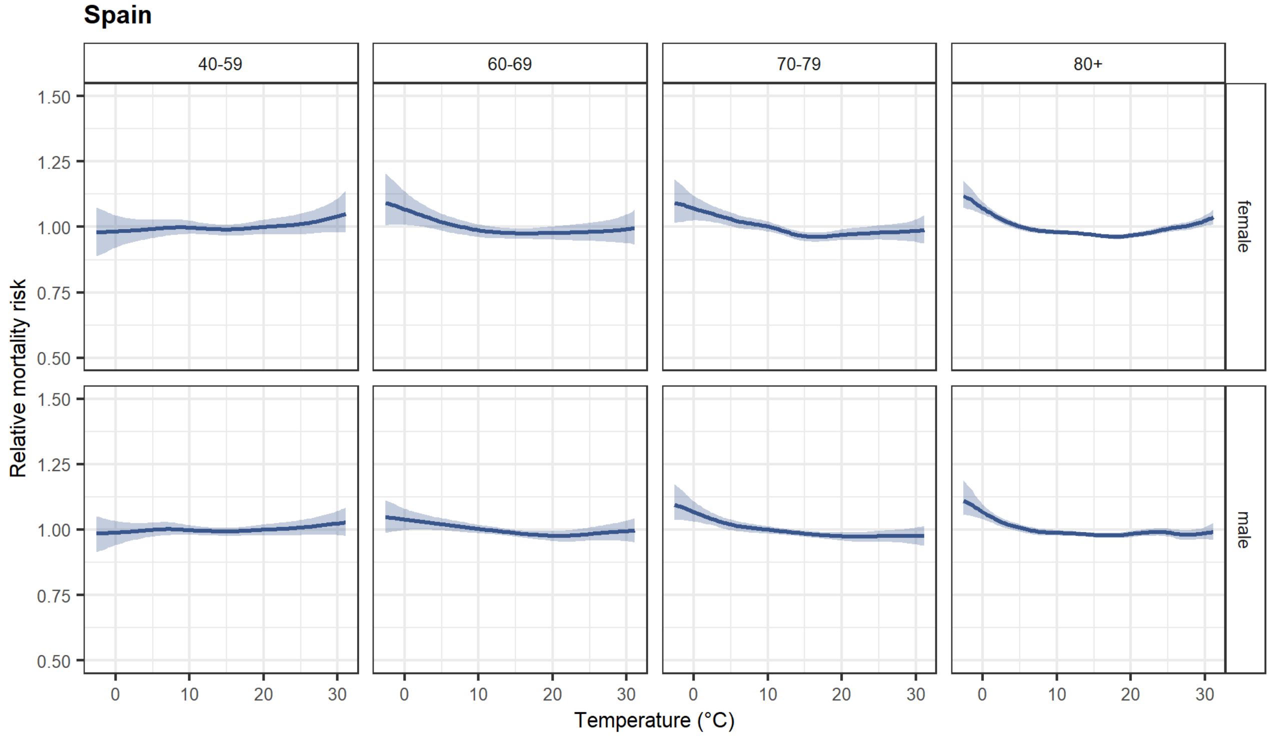

Data are presented as median relative mortality risk (the blue line) and 95% Credible Intervals (shaded blue areas).

Supplementary Figure 6: Relative mortality risk of the effect of temperature during 2015 and 2020 by age and sex groups in Switzerland.

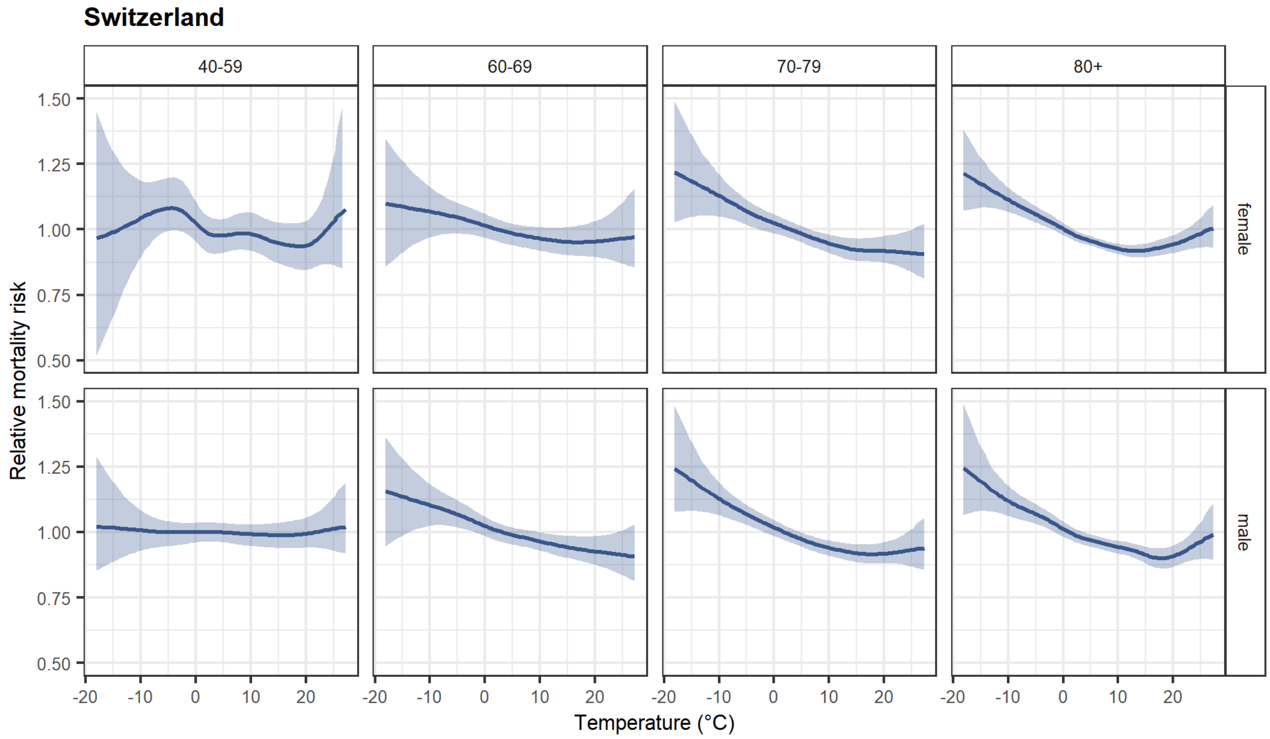

120 Data are presented as median relative mortality risk (the blue line) and 95% Credible Intervals (shaded blue
121 areas).

Supplementary Figure 7: Weekly trends for all-cause total number of deaths by gender and age in England. The blue curve represents the posterior mean predicted from the 2015–2019 model, while the shaded blue ribbon describes the posterior 95% interval. The black line shows the observed number of deaths for 2020.

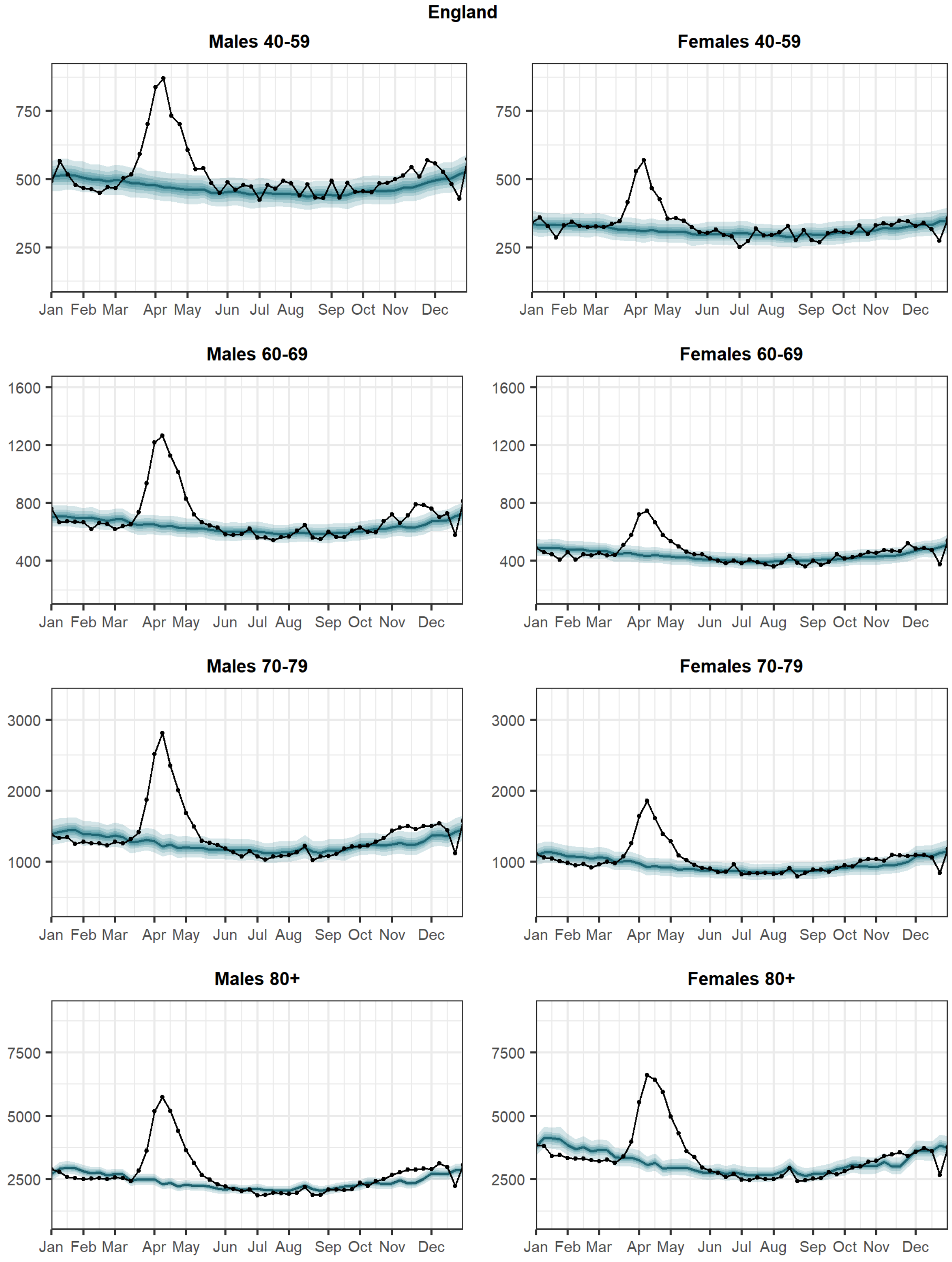

Supplementary Figure 8: Weekly trends for all-cause total number of deaths by gender and age in Greece. The blue curve represents the posterior mean predicted from the 2015–2019 model, while the shaded blue ribbon describes the posterior 95% interval. The black line shows the observed number of deaths for 2020.

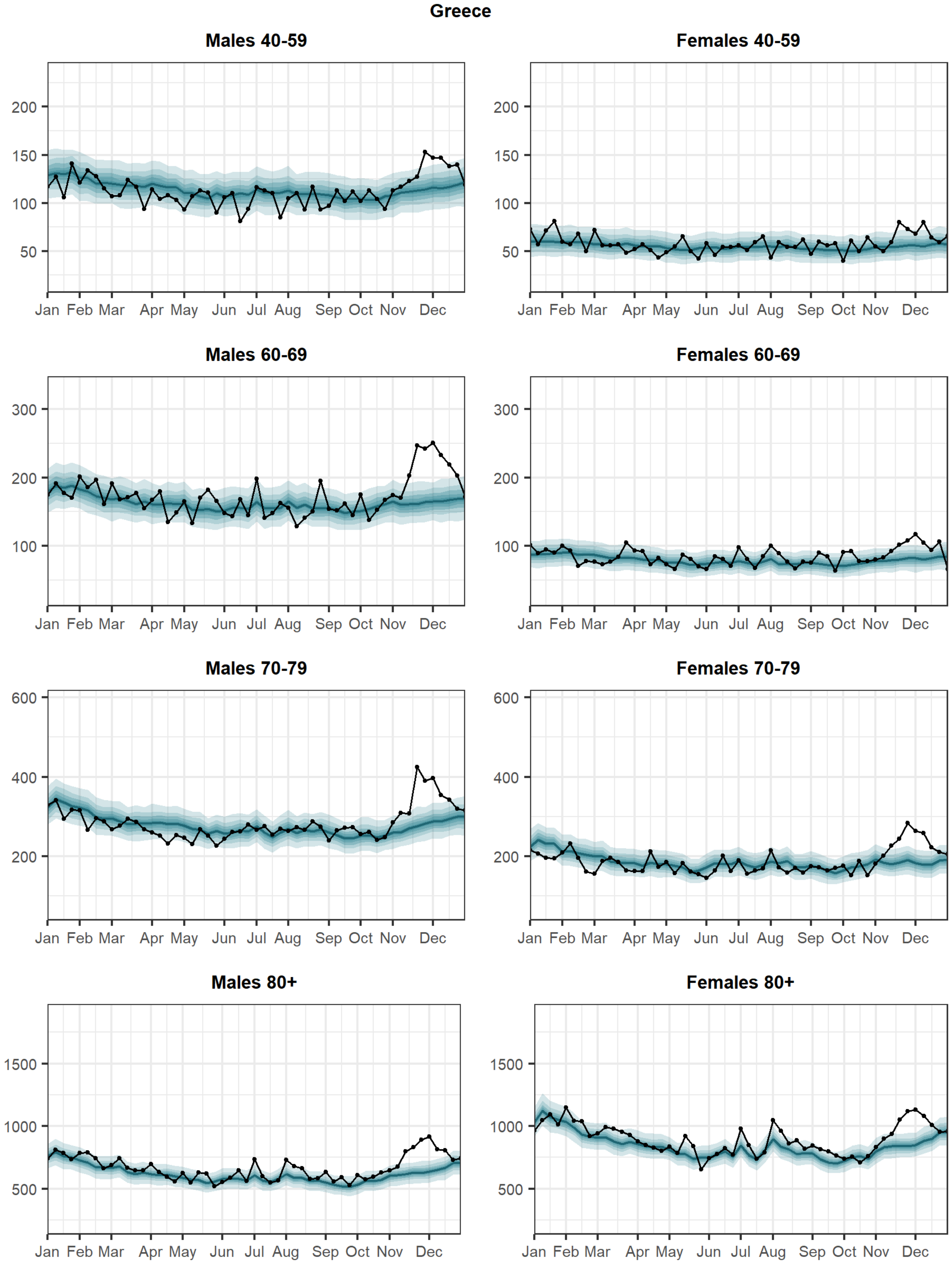

Supplementary Figure 9: Weekly trends for all-cause total number of deaths by gender and age in Italy. The blue curve represents the posterior mean predicted from the 2015–2019 model, while the shaded blue ribbon describes the posterior 95% interval. The black line shows the observed number of deaths for 2020.

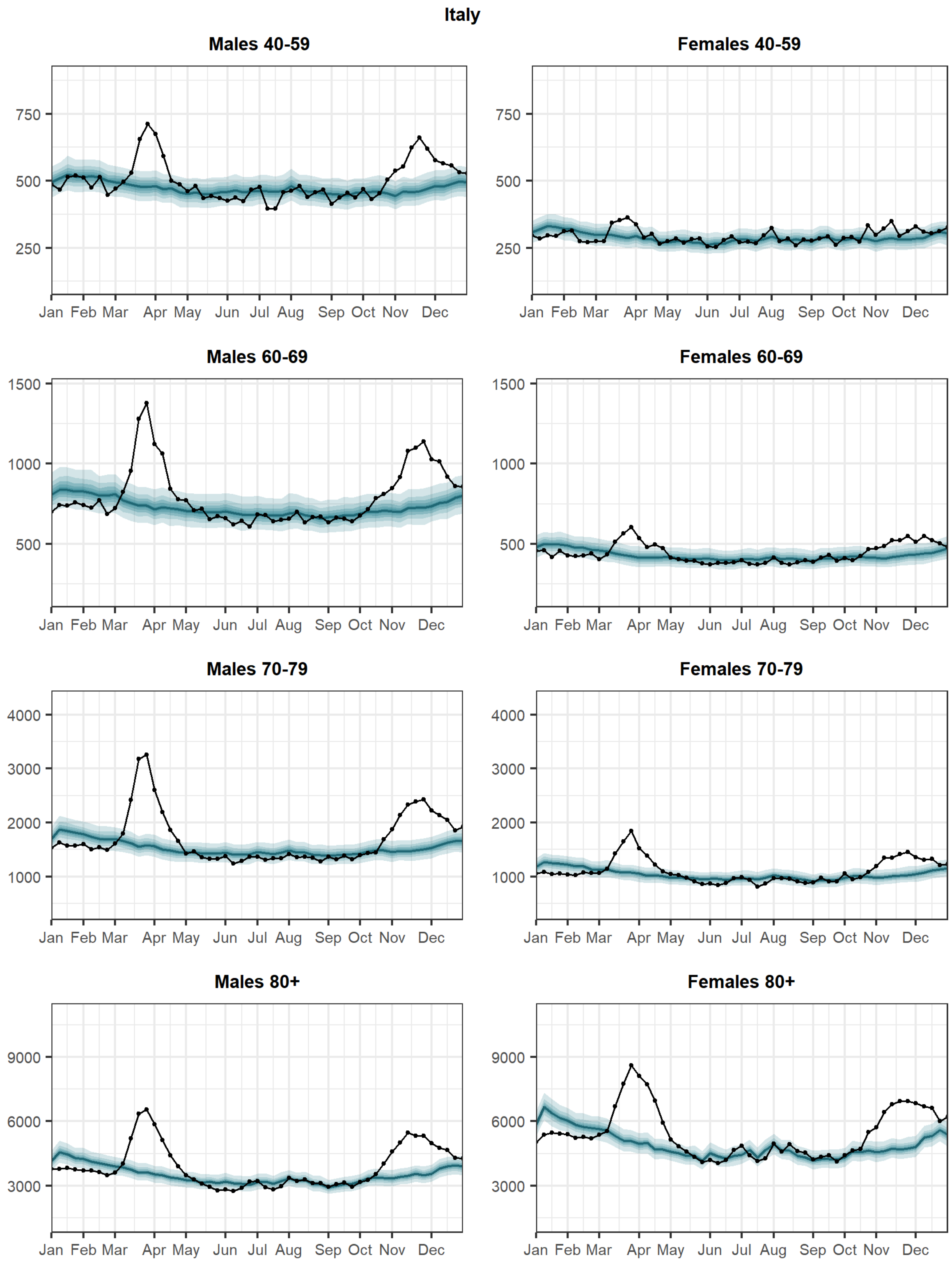

Supplementary Figure 10: Weekly trends for all-cause total number of deaths by gender and age in Spain. The blue curve represents the posterior mean predicted from the 2015–2019 model, while the shaded blue ribbon describes the posterior 95% interval. The black line shows the observed number of deaths for 2020.

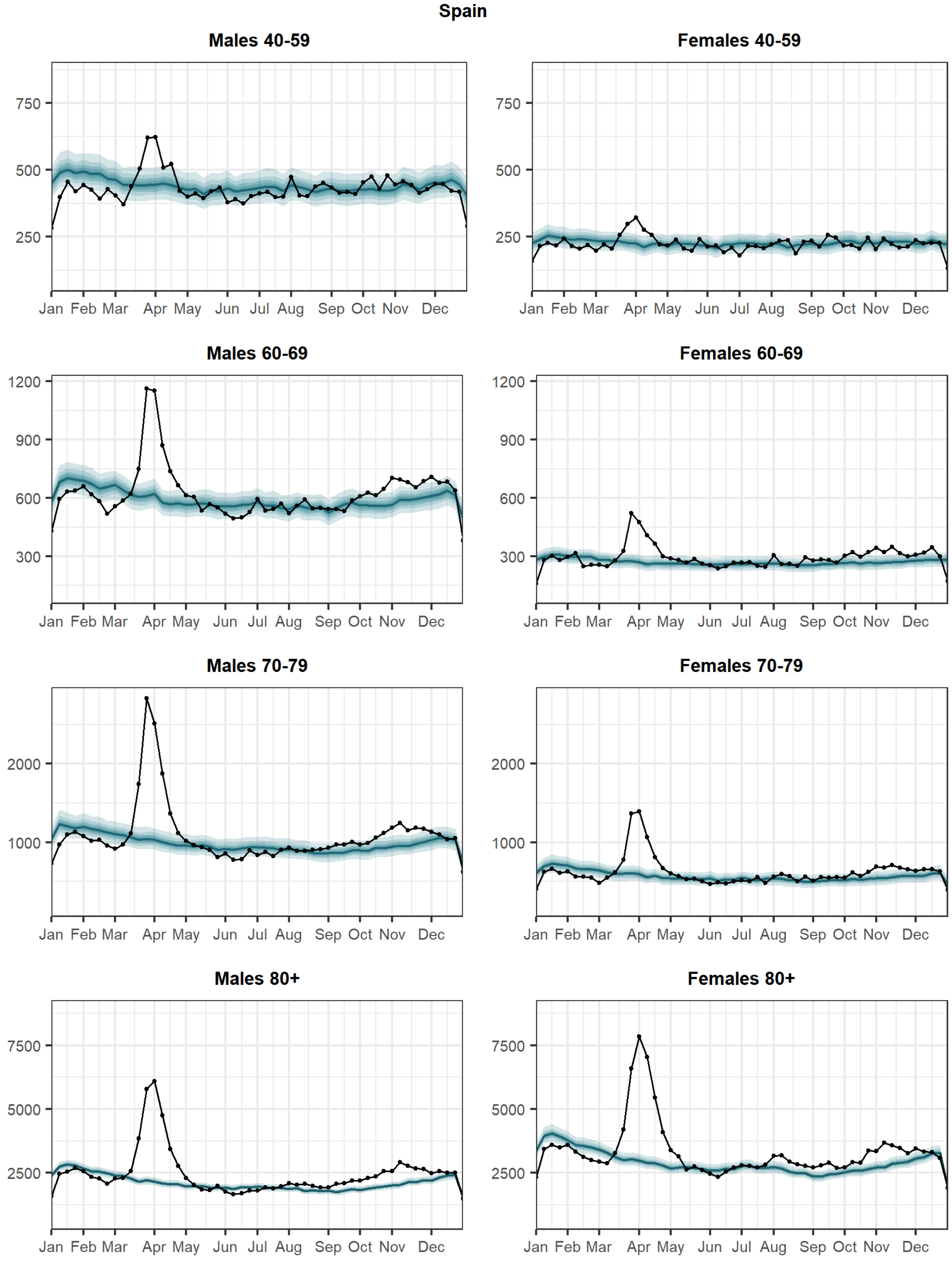

Supplementary Figure 11: Weekly trends for all-cause total number of deaths by gender and age in Switzerland. The blue curve represents the posterior mean predicted from the 2015–2019 model, while the shaded blue ribbon describes the posterior 95% interval. The black line shows the observed number of deaths for 2020.

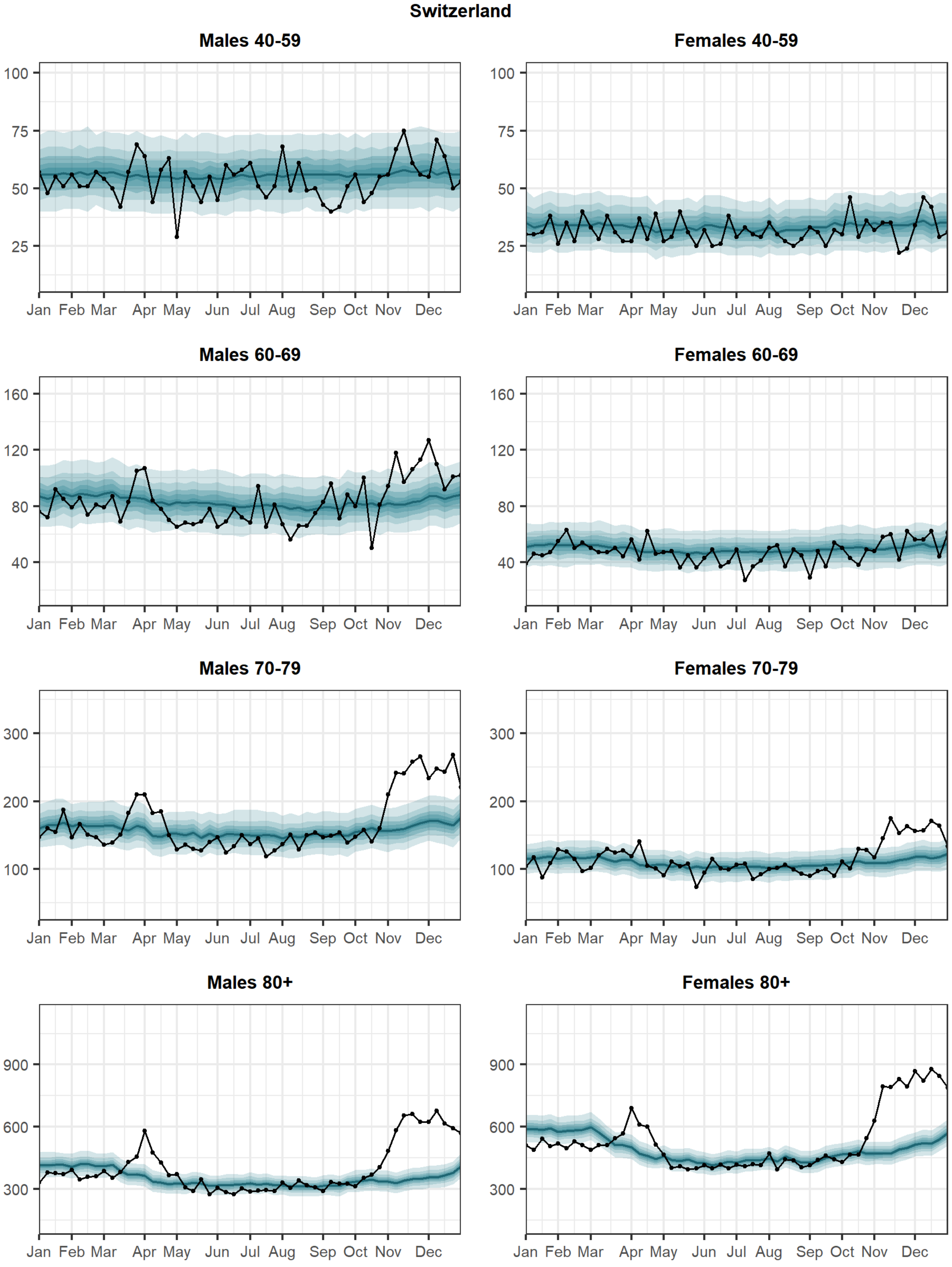

Supplementary Figure 12: Relative excess mortality during 2020 by NUTS3 regions in England stratified by age and sex.

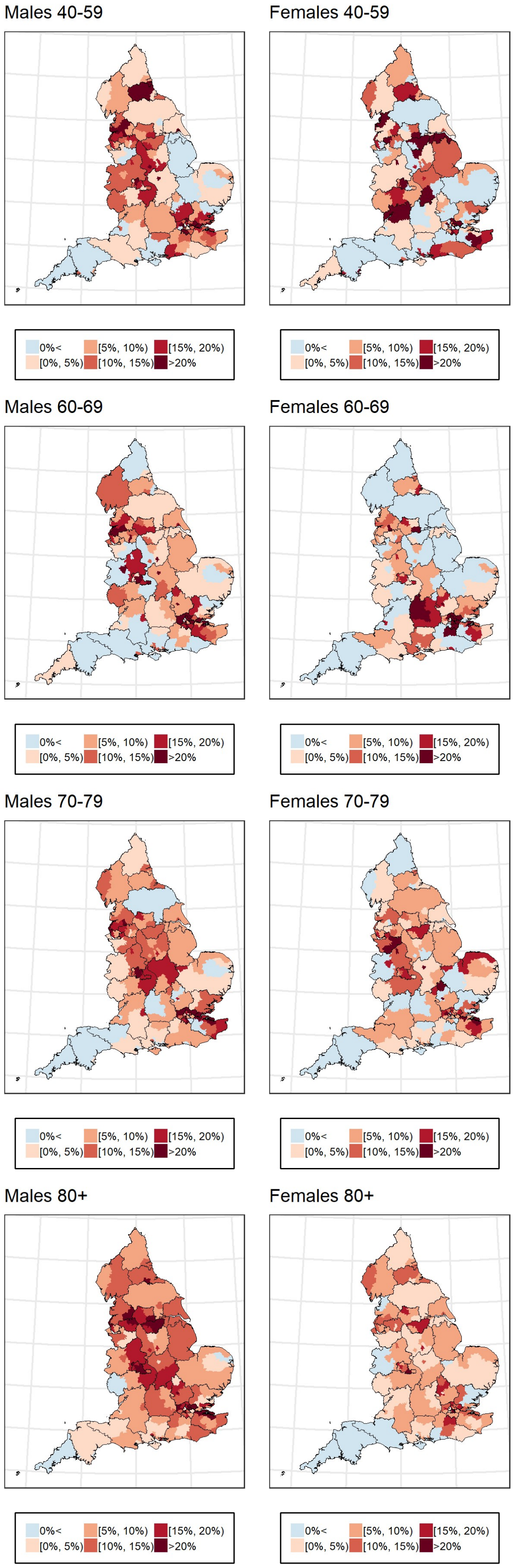

Supplementary Figure 13: Posterior probability that the relative excess mortality is larger than zero during 2020 by NUTS3 regions in England stratified by age and sex.

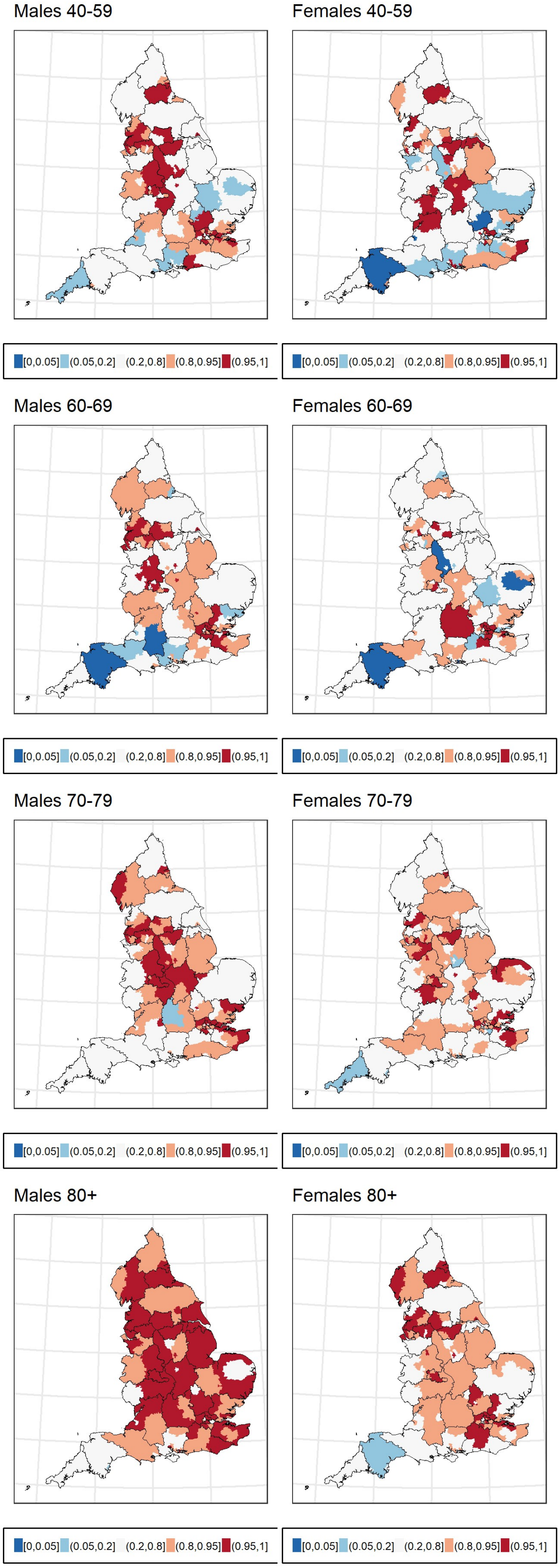

Supplementary Figure 14: Relative excess mortality during 2020 by NUTS3 regions in Greece stratified by age and sex.

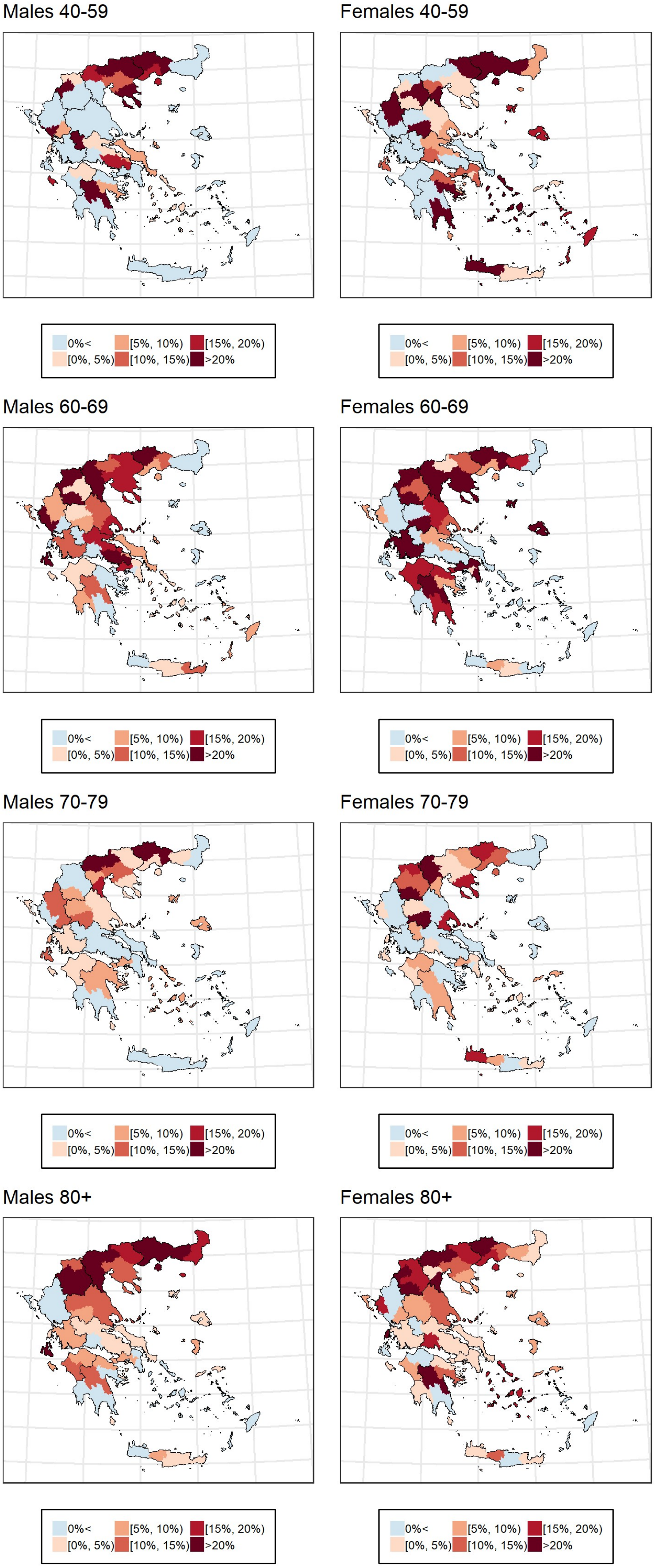

Supplementary Figure 15: Posterior probability that the relative excess mortality is larger than zero during 2020 by NUTS3 regions in Greece stratified by age and sex.

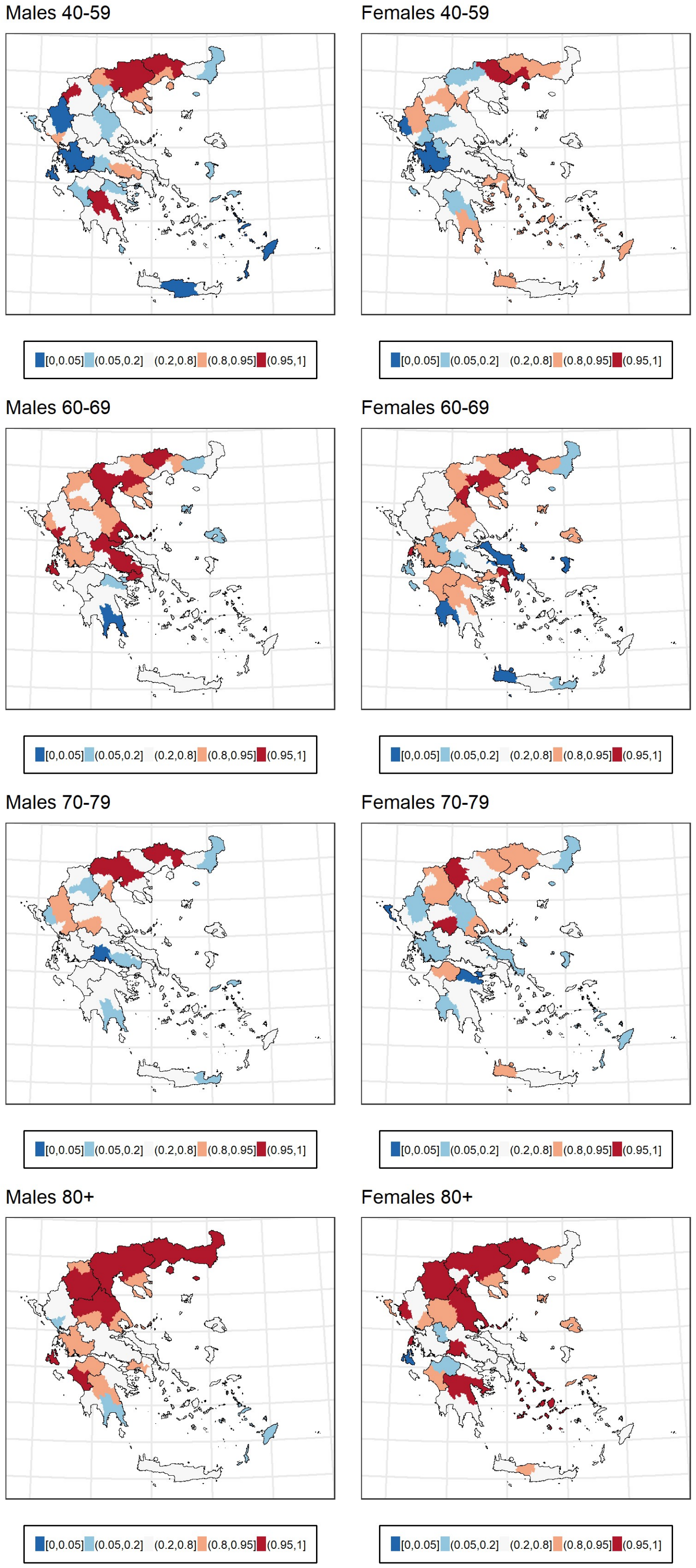

Supplementary Figure 16: Relative excess mortality during 2020 by NUTS3 regions in Italy stratified by age and sex.

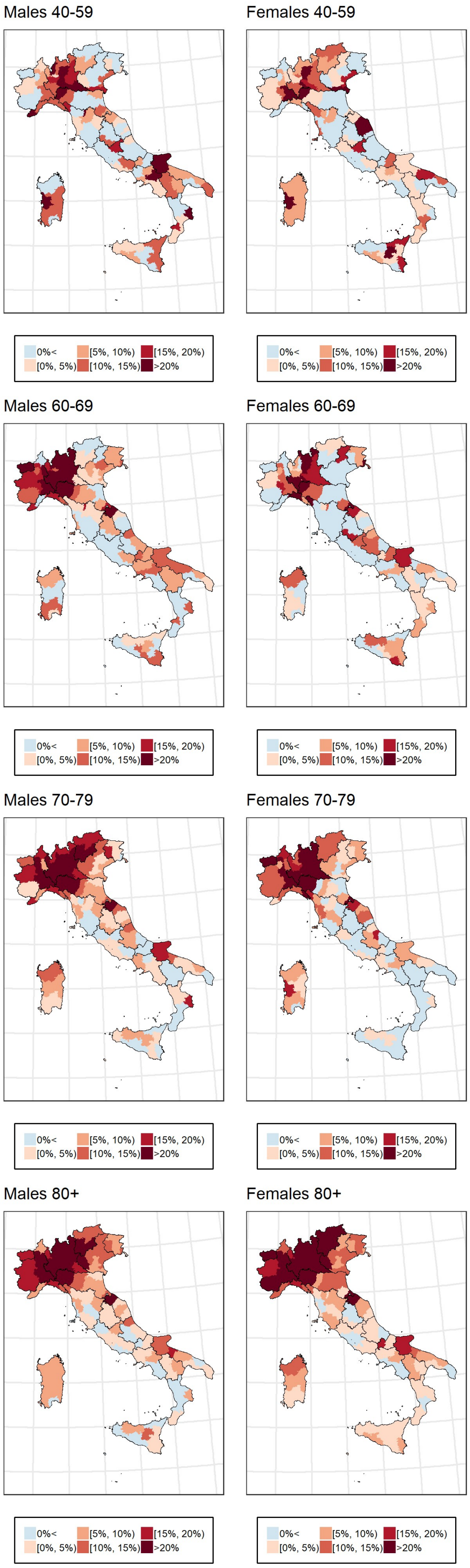

Supplementary Figure 17: Posterior probability that the relative excess mortality is larger than zero during 2020 by NUTS3 regions in Italy stratified by age and sex.

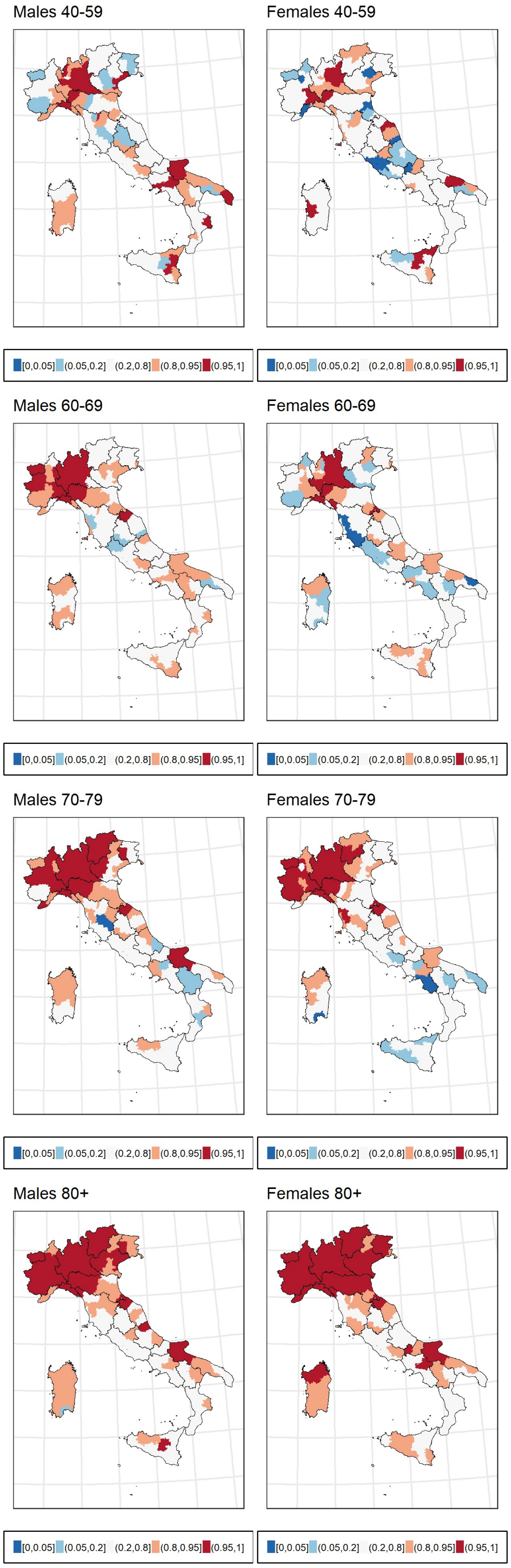

Supplementary Figure 18: Relative excess mortality during 2020 by NUTS3 regions in Spain stratified by age and sex.

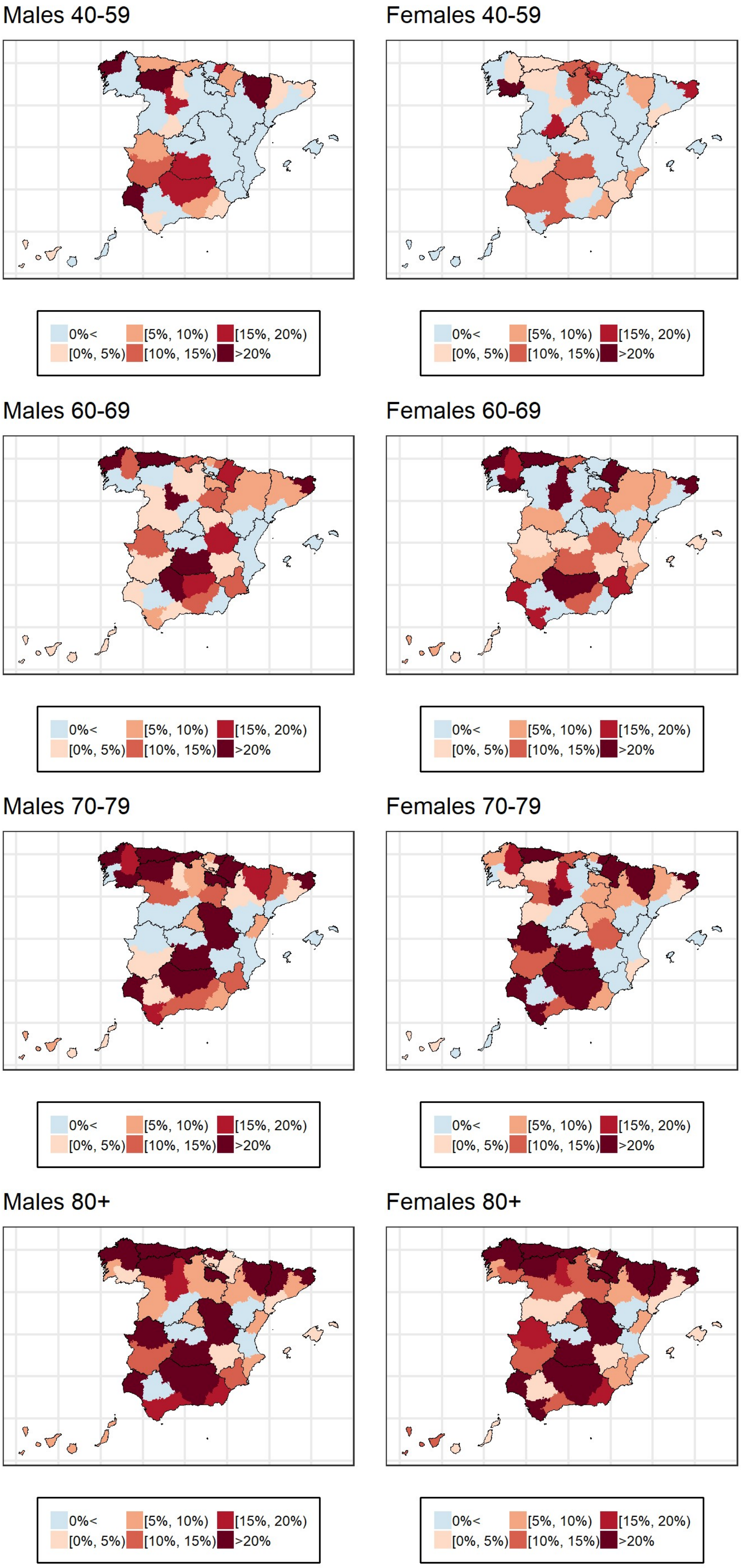

Supplementary Figure 19: Posterior probability that the relative excess mortality is larger than zero during 2020 by NUTS3 regions in Spain stratified by age and sex.

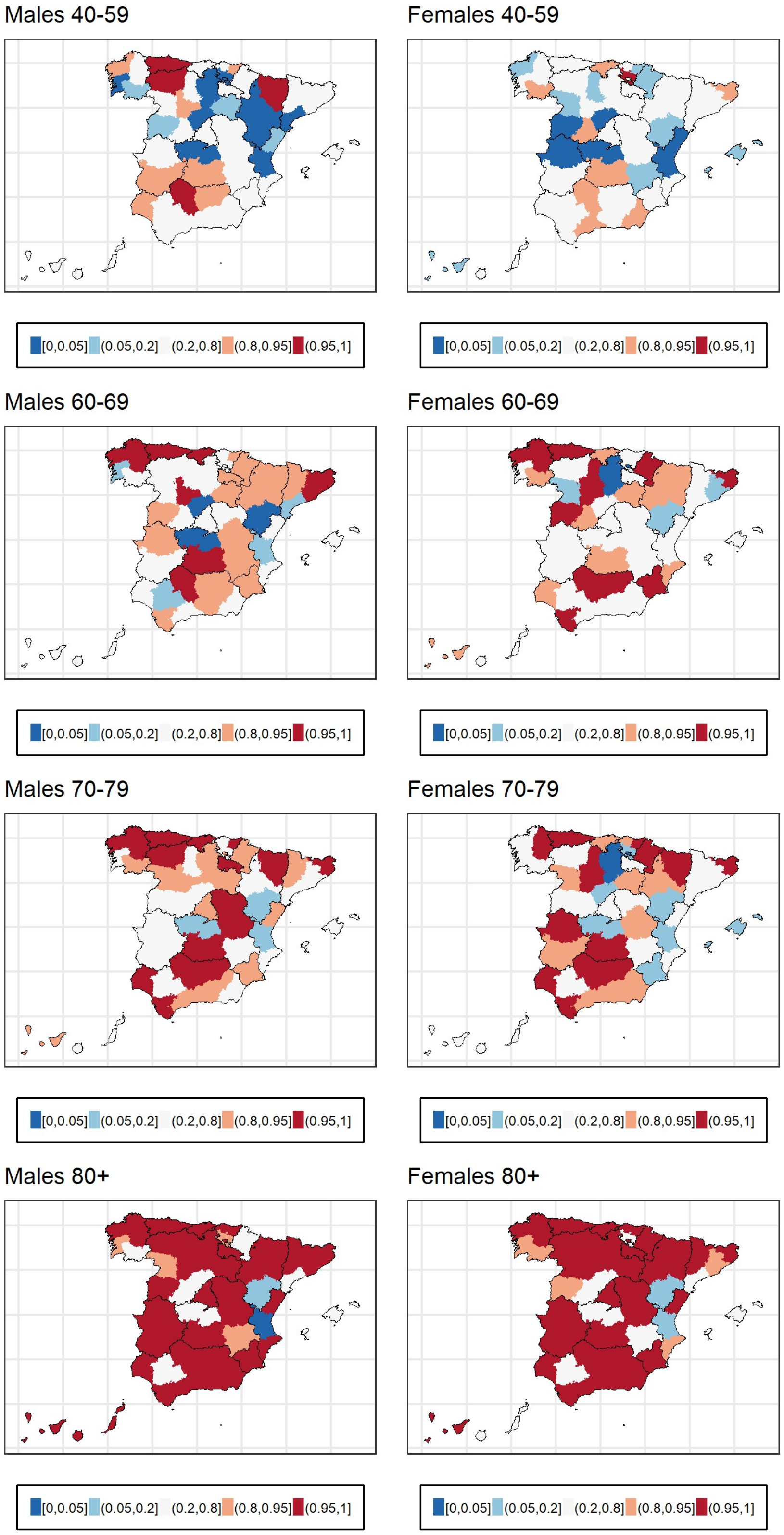

Supplementary Figure 20: Relative excess mortality during 2020 by NUTS3 regions in Switzerland stratified by age and sex.

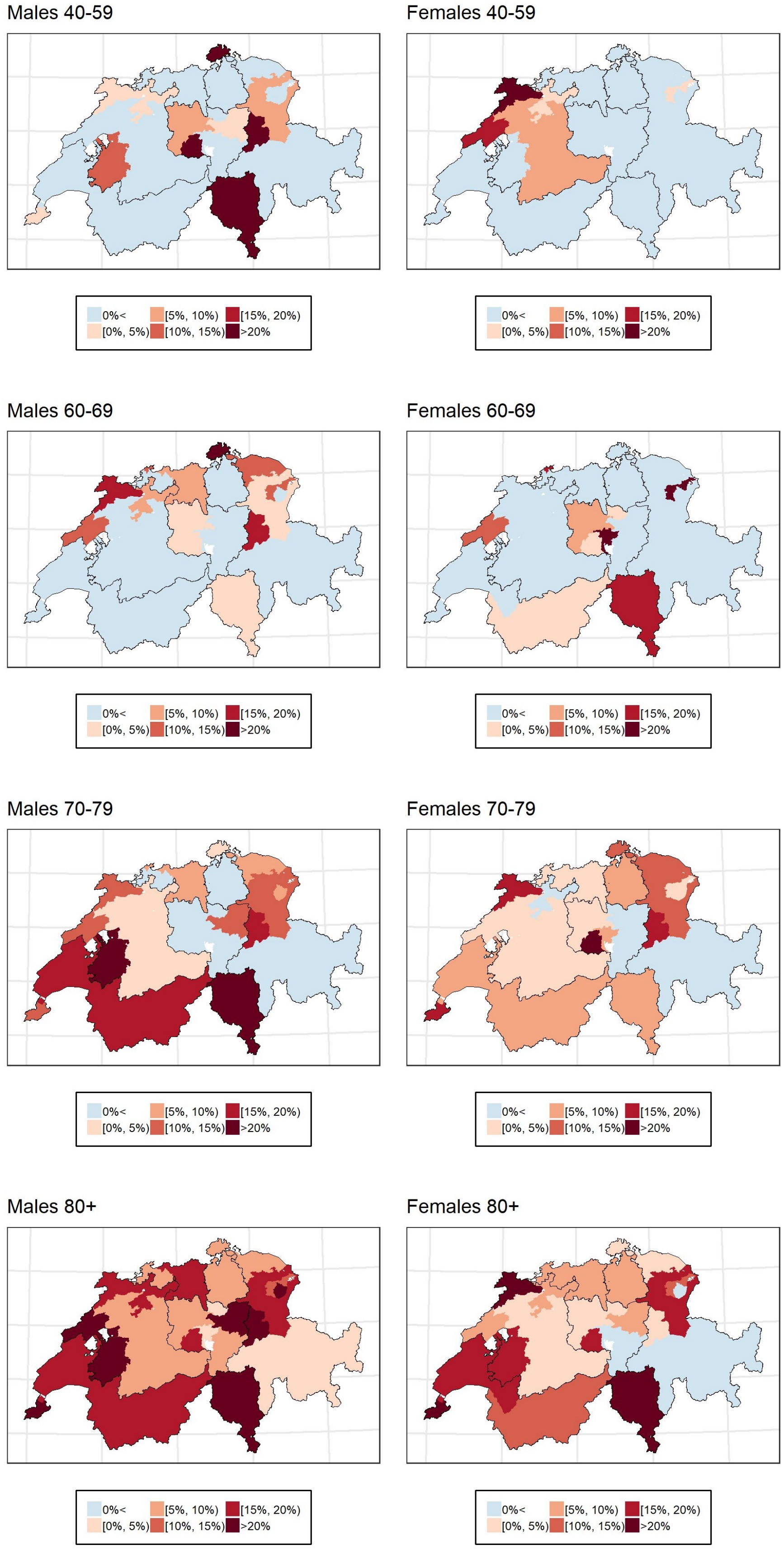

Supplementary Figure 21: Posterior probability that the relative excess mortality is larger than zero during 2020 by NUTS3 regions in Switzerland stratified by age and sex.

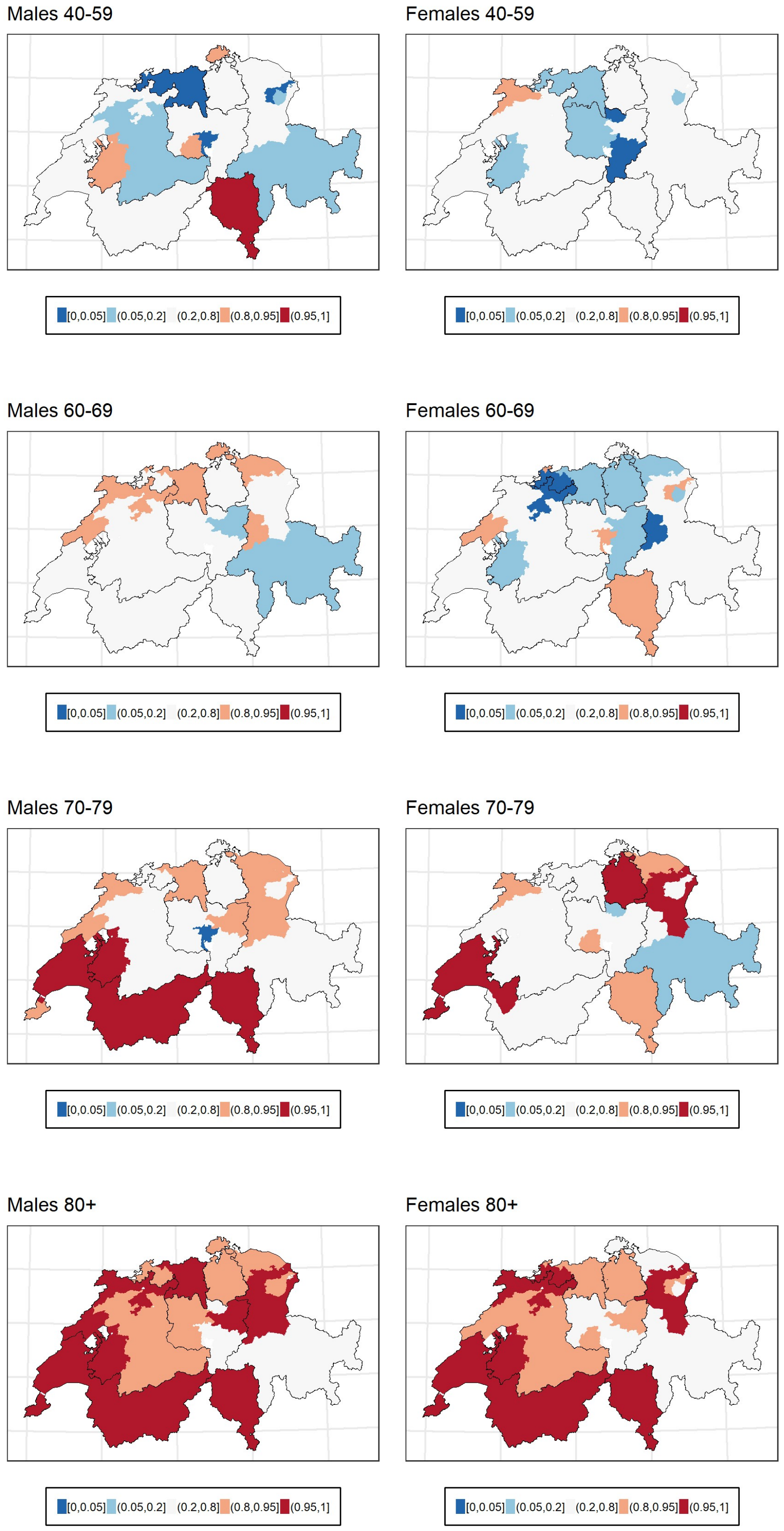

Supplementary Figure 22: Relative excess mortality during January-June 2020 by NUTS3 regions in England stratified by age and sex.

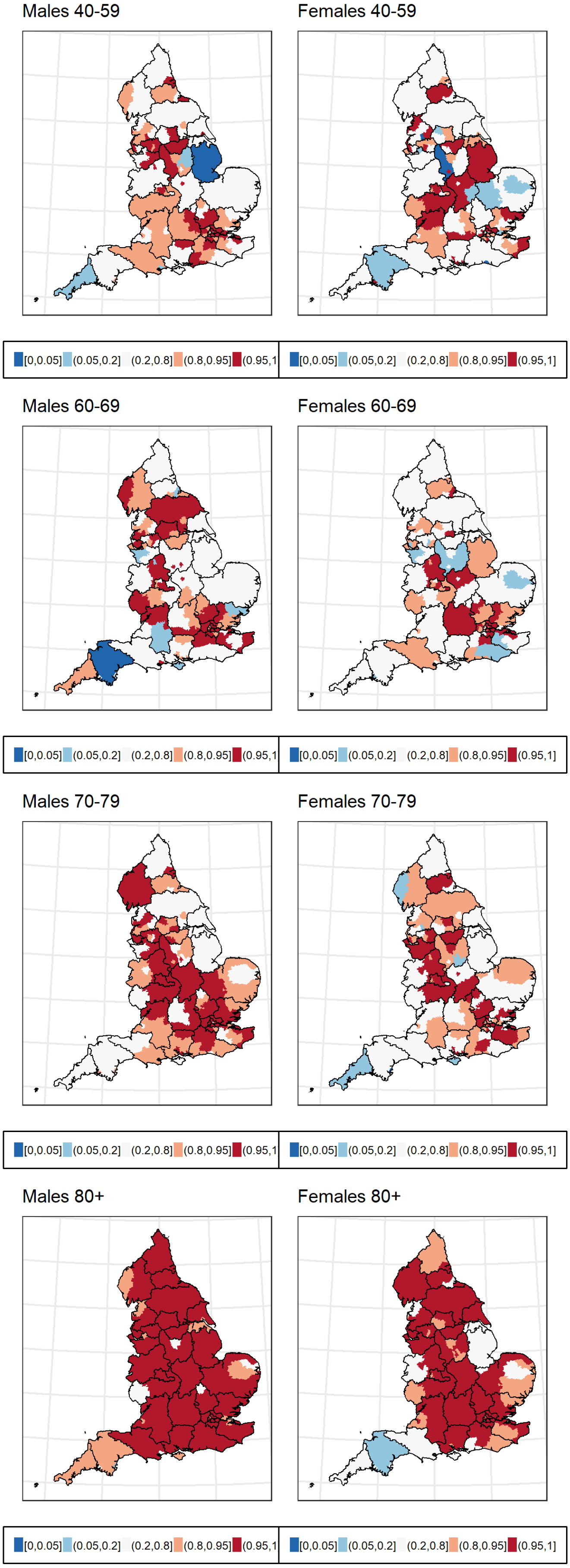

Supplementary Figure 23: Relative excess mortality during July-December 2020 by NUTS3 regions in England stratified by age and sex.

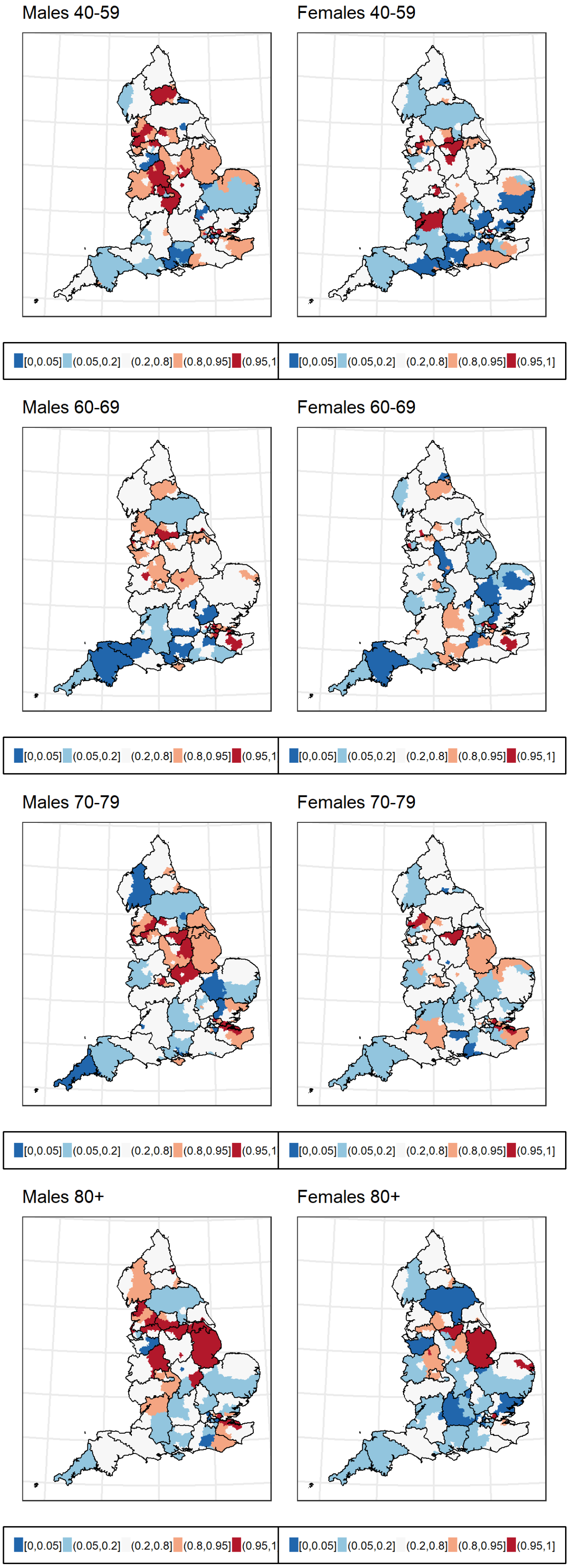

Supplementary Figure 24: Posterior probability that the relative excess mortality is larger than zero during January-June 2020 by NUTS3 regions in England stratified by age and sex.

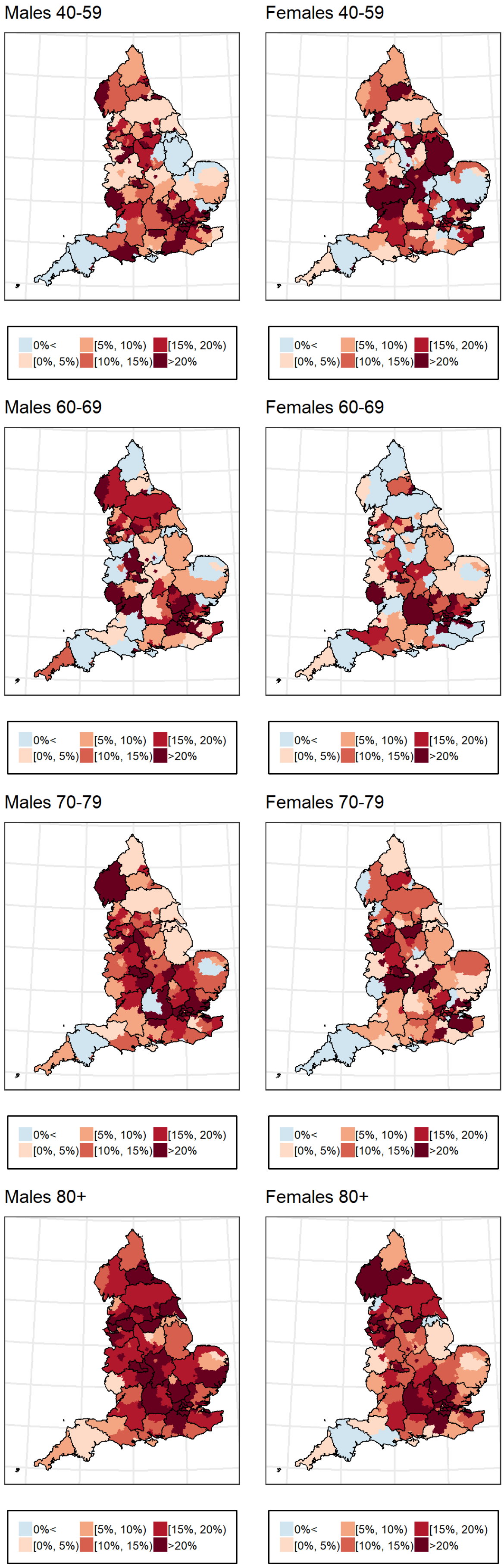

Supplementary Figure 25: Posterior probability that the relative excess mortality is larger than zero during July-December 2020 by NUTS3 regions in England stratified by age and sex.

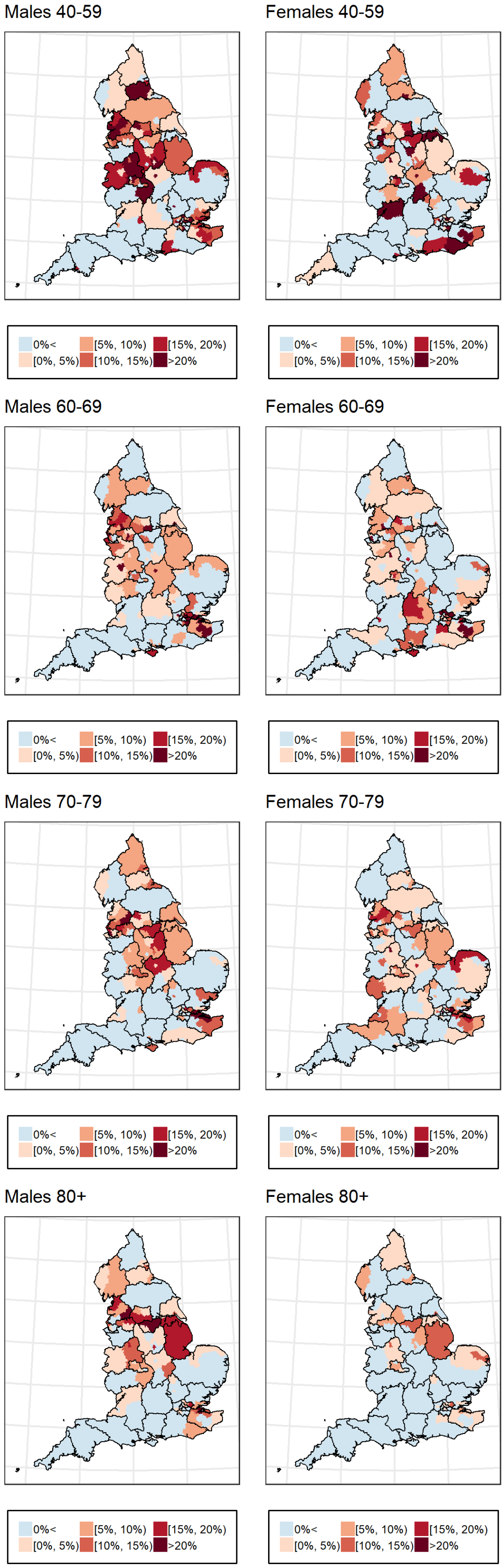

Supplementary Figure 26: Relative excess mortality during January-June 2020 by NUTS3 regions in Greece stratified by age and sex.

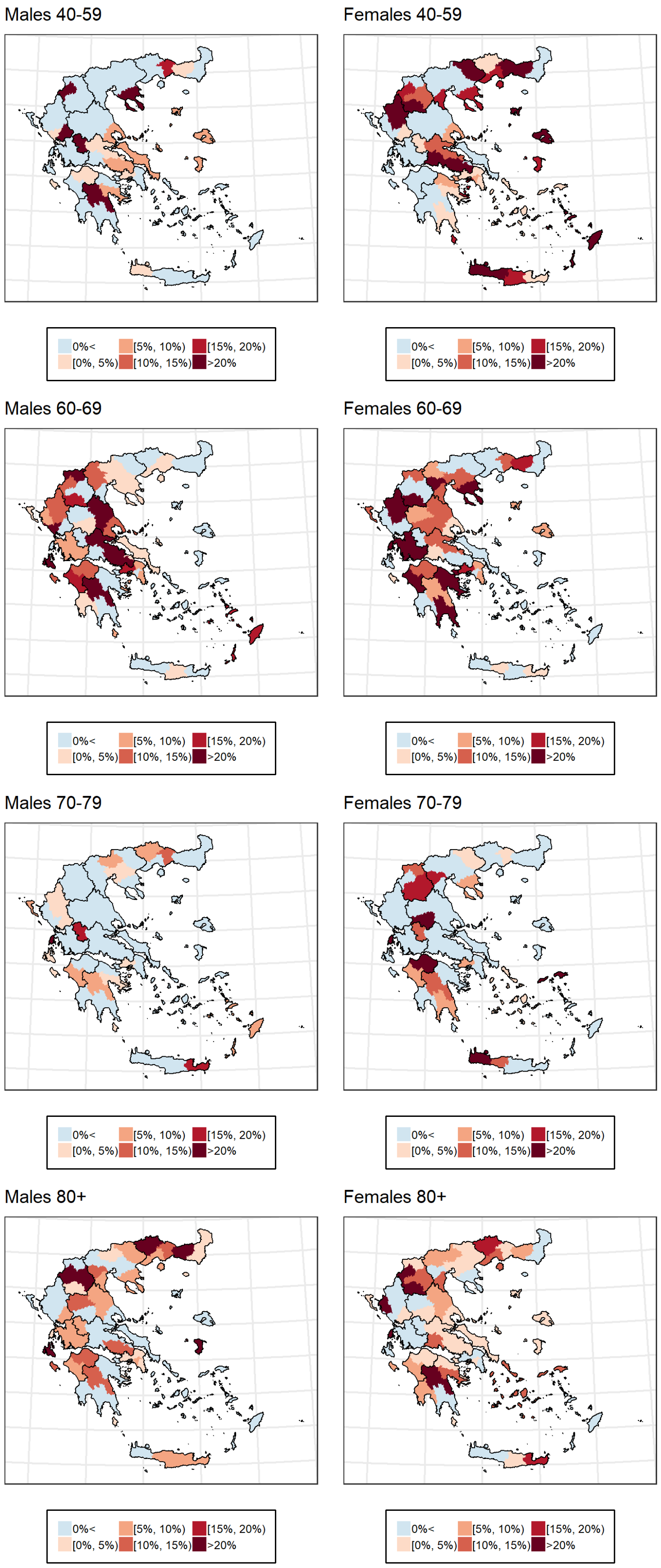

Supplementary Figure 27: Relative excess mortality during July-December 2020 by NUTS3 regions in Greece stratified by age and sex.

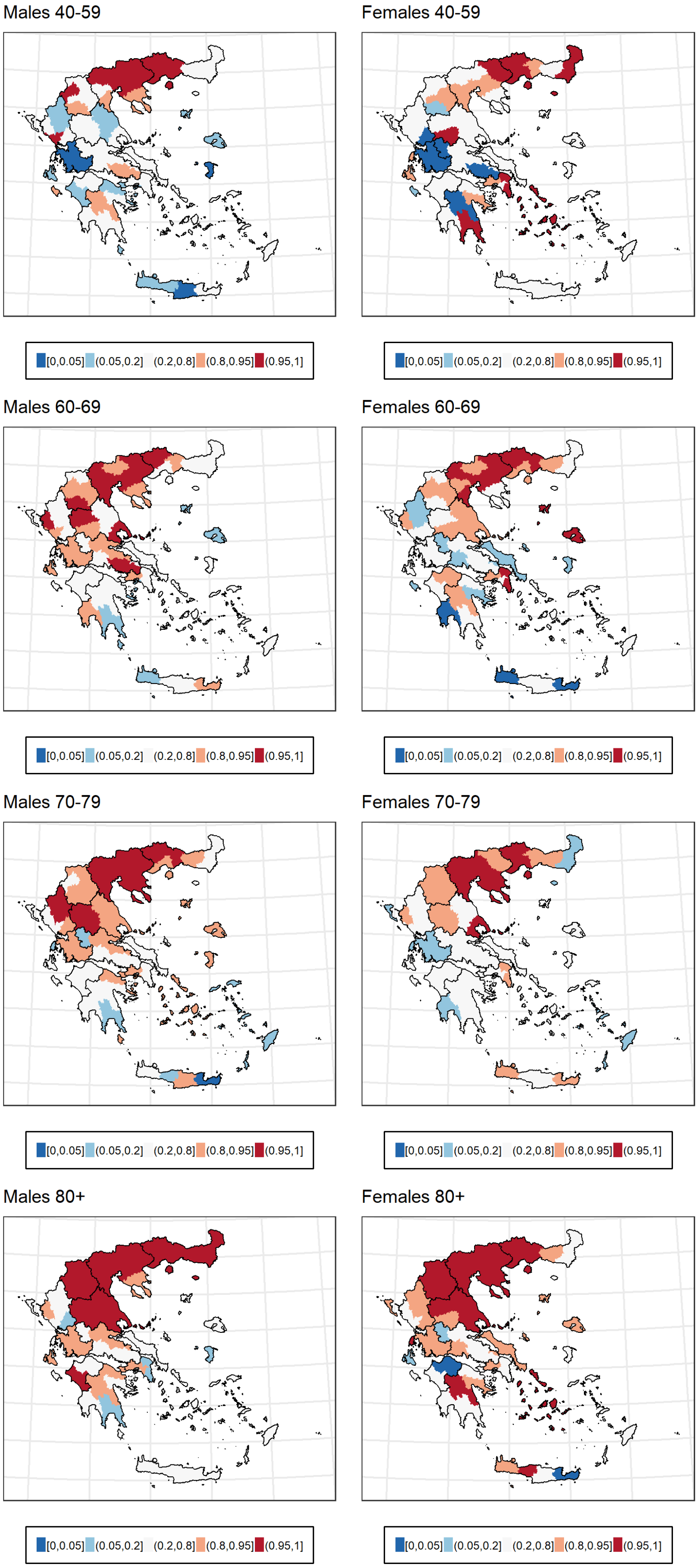

Supplementary Figure 28: Posterior probability that the relative excess mortality is larger than zero during January-June 2020 by NUTS3 regions in Greece stratified by age and sex.

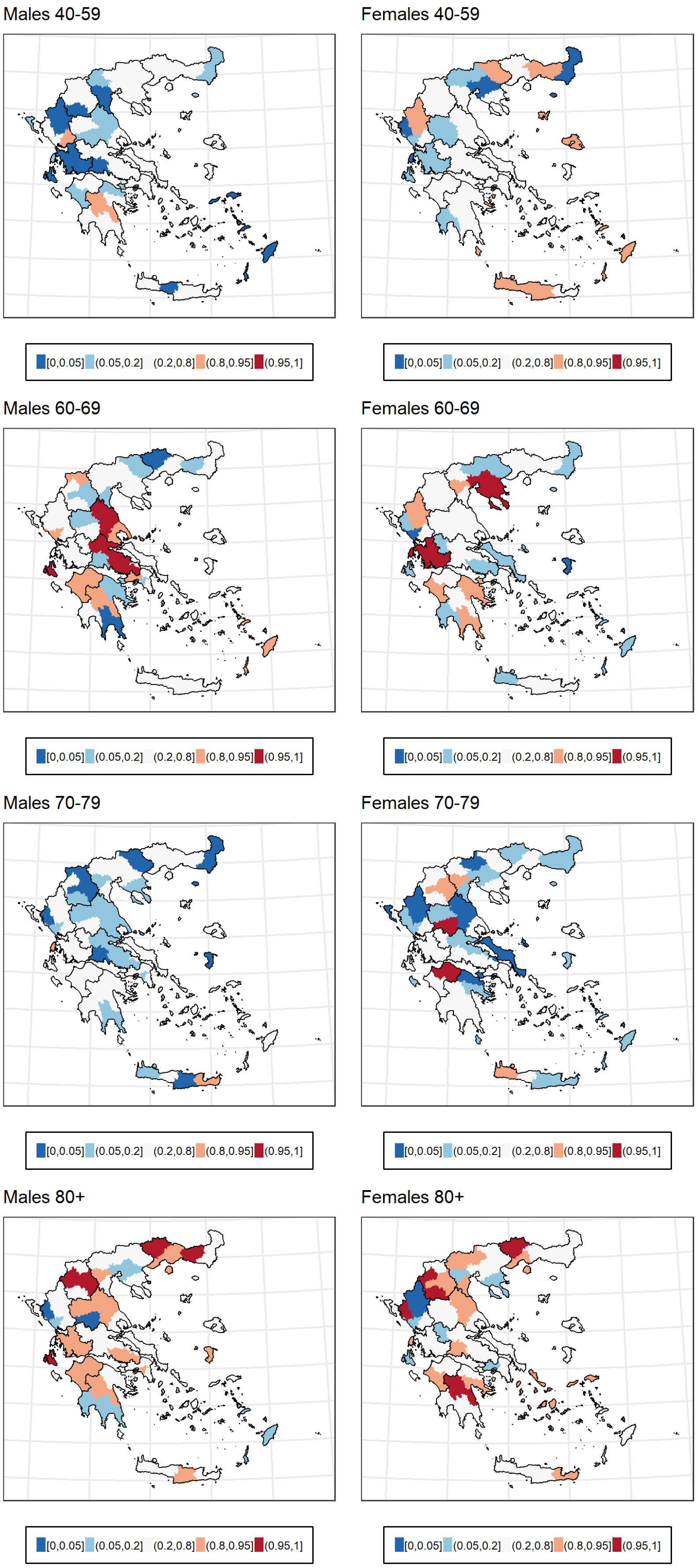

Supplementary Figure 29: Posterior probability that the relative excess mortality is larger than zero during July-December 2020 by NUTS3 regions in Greece stratified by age and sex.

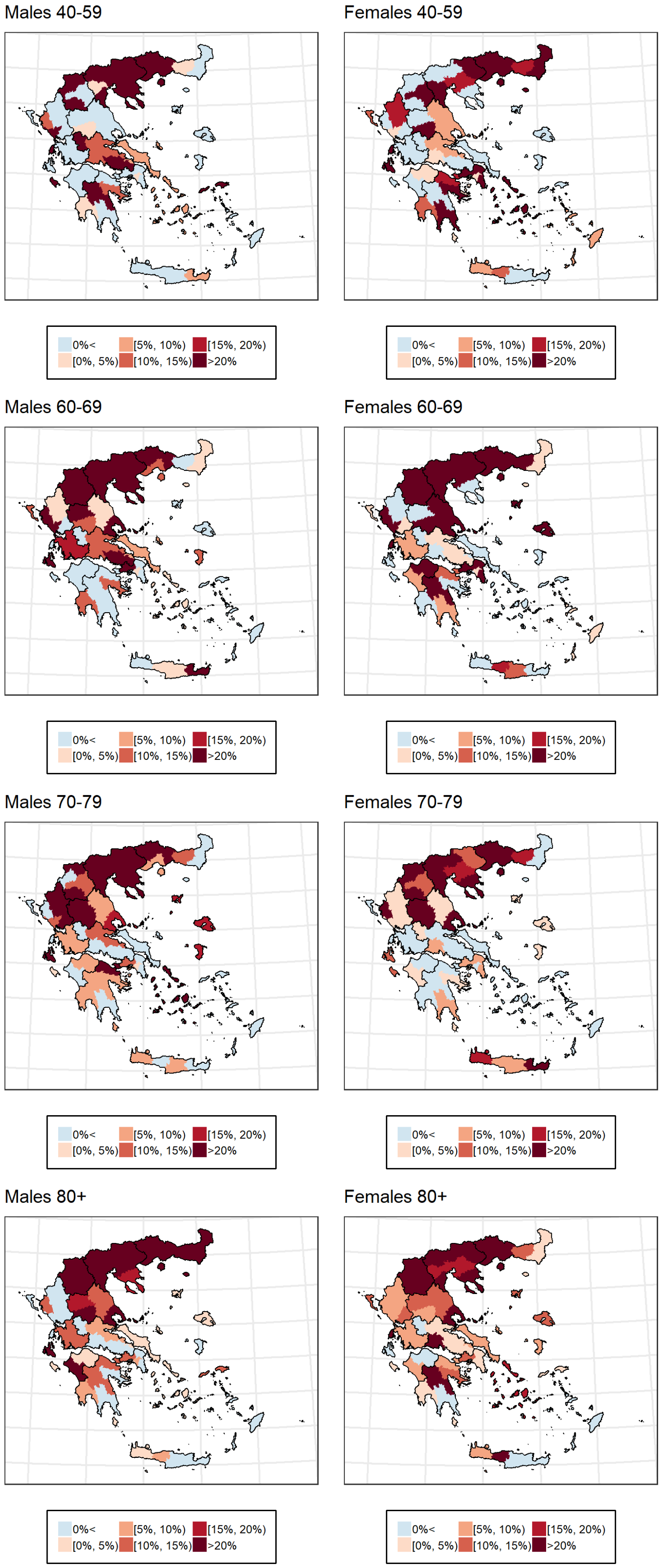

Supplementary Figure 30: Relative excess mortality during January-June 2020 by NUTS3 regions in Italy stratified by age and sex.

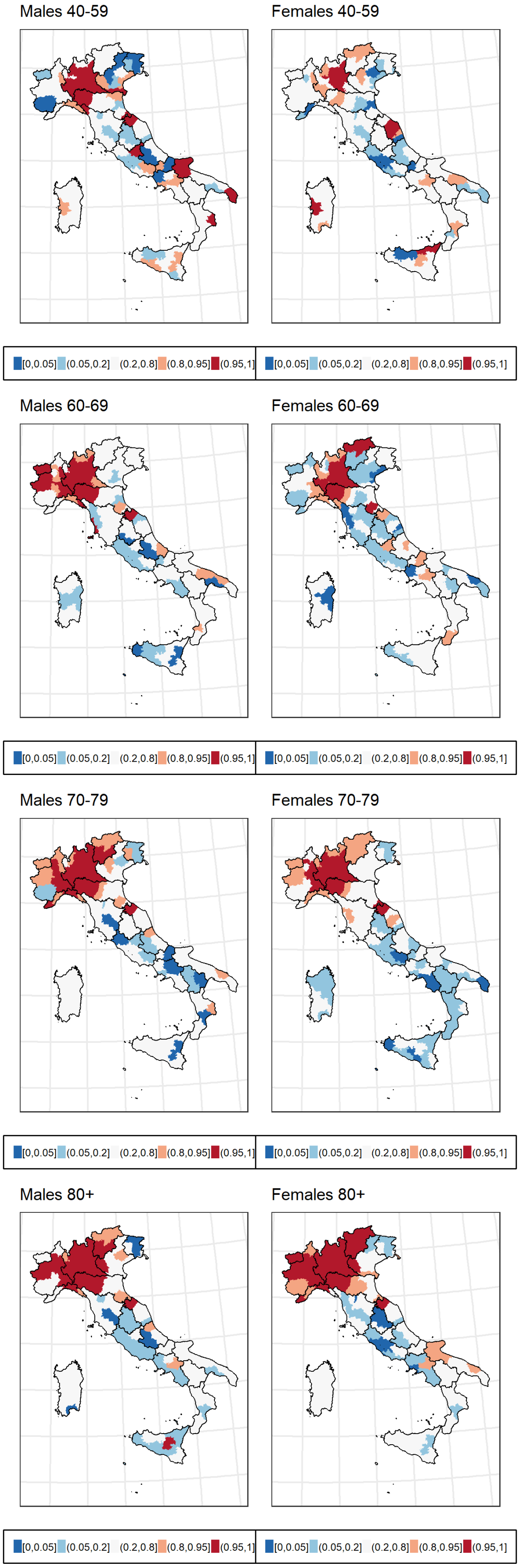

Supplementary Figure 31: Relative excess mortality during July-December 2020 by NUTS3 regions in Italy stratified by age and sex.

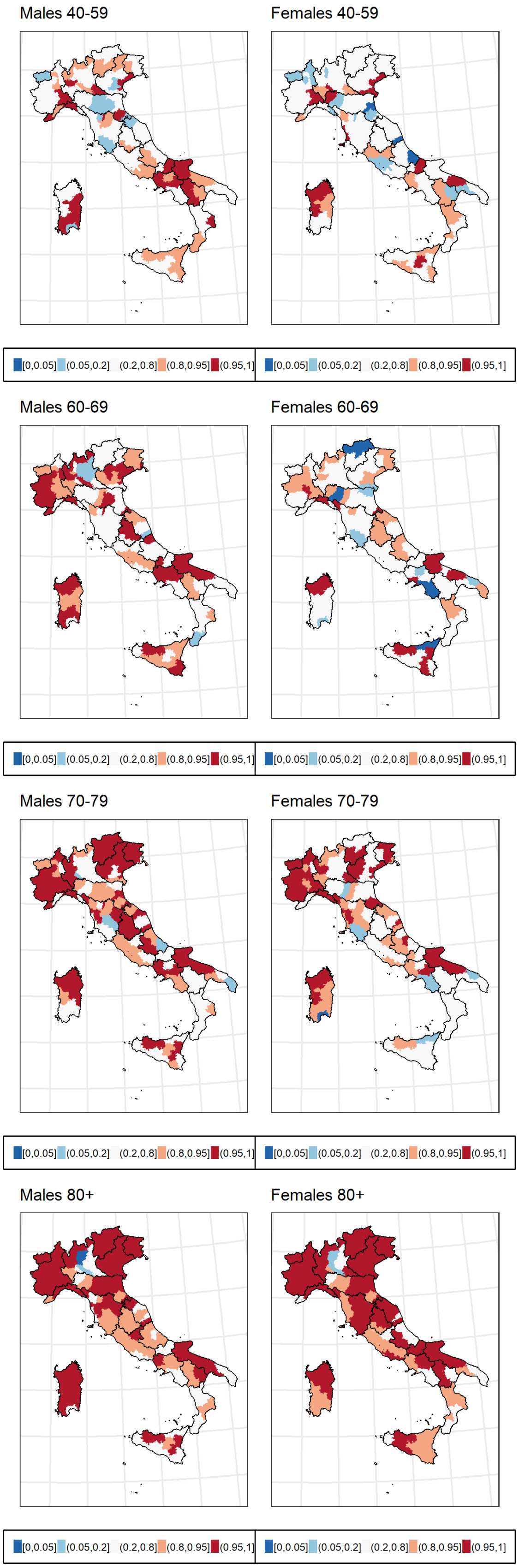

Supplementary Figure 32: Posterior probability that the relative excess mortality is larger than zero during January-June 2020 by NUTS3 regions in Italy stratified by age and sex.

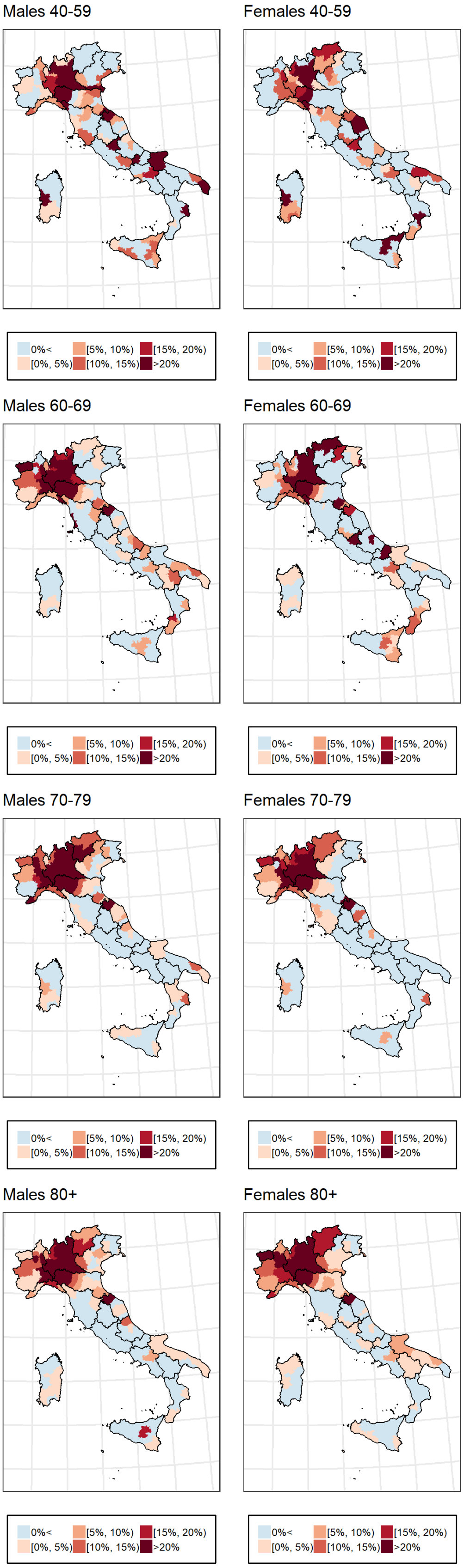

Supplementary Figure 33: Posterior probability that the relative excess mortality is larger than zero during July-December 2020 by NUTS3 regions in Italy stratified by age and sex.

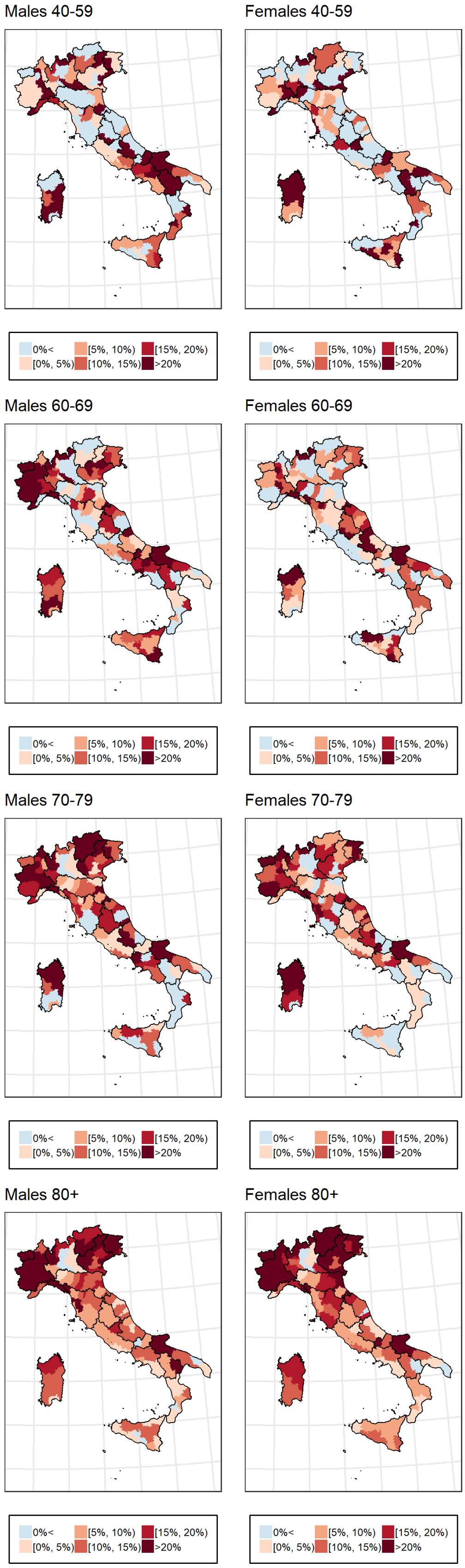

Supplementary Figure 34: Relative excess mortality during January-June 2020 by NUTS3 regions in Spain stratified by age and sex.

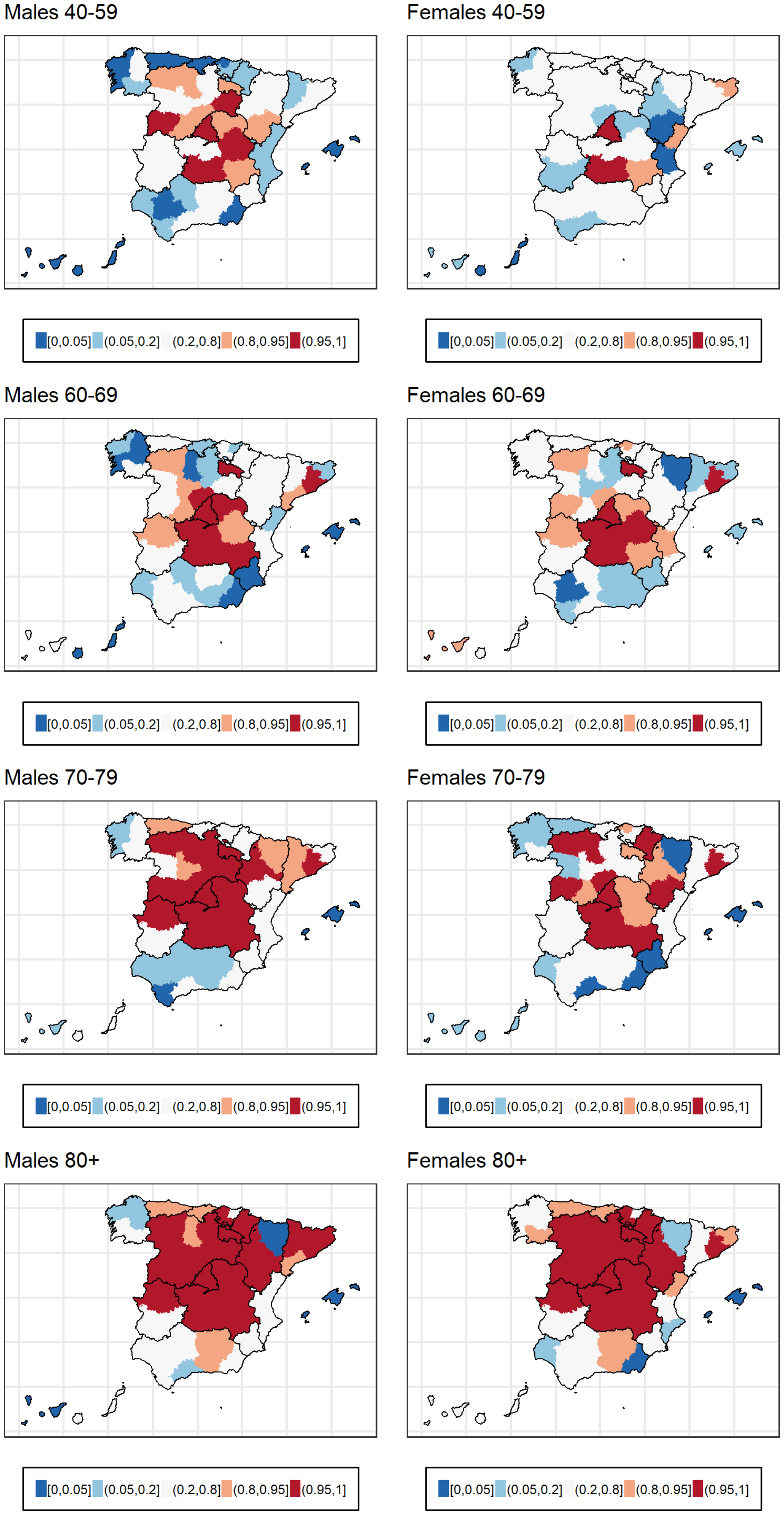

Supplementary Figure 35: Relative excess mortality during July-December 2020 by NUTS3 regions in Spain stratified by age and sex.

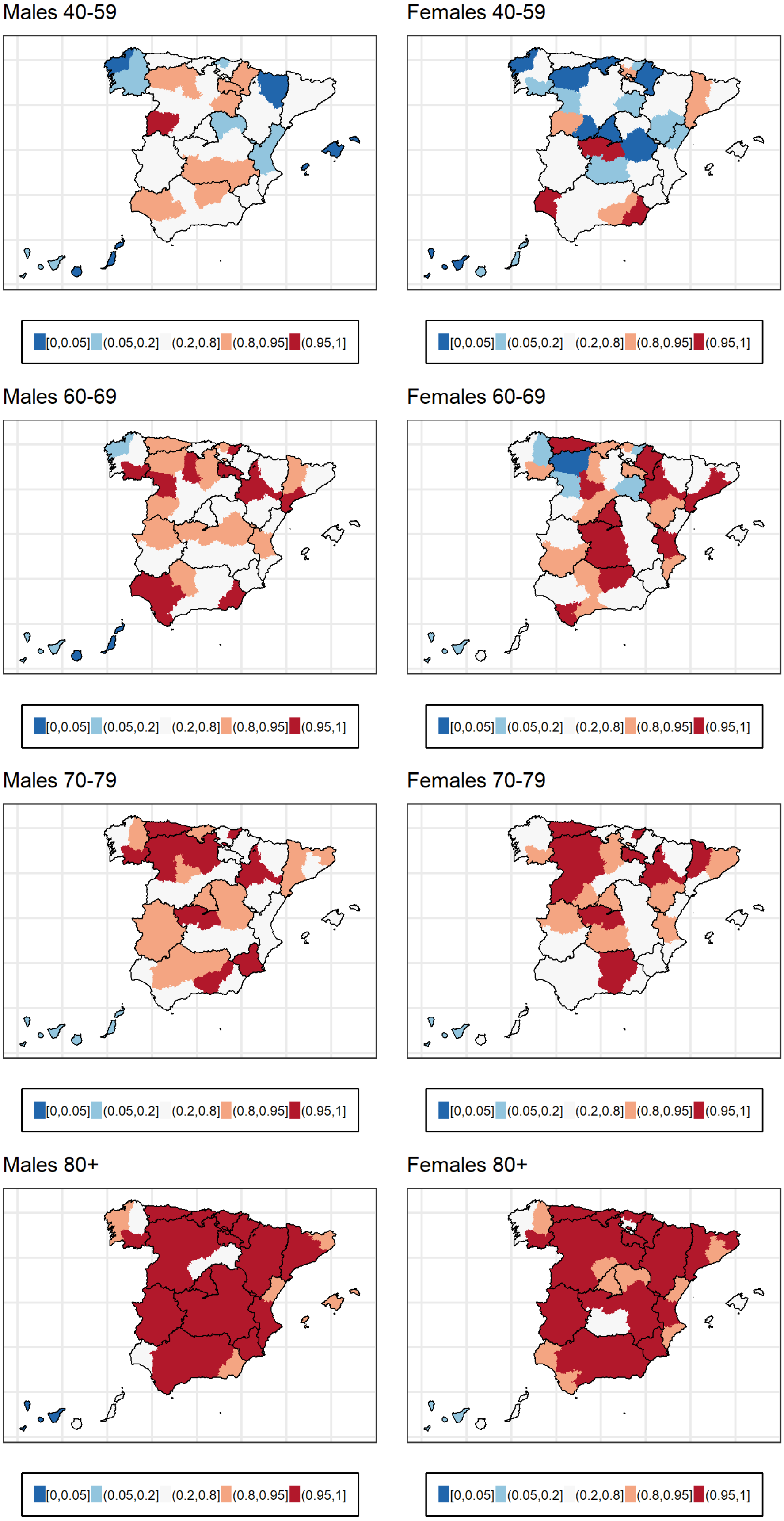

Supplementary Figure 36: Posterior probability that the relative excess mortality is larger than zero during January-June 2020 by NUTS3 regions in Spain stratified by age and sex.

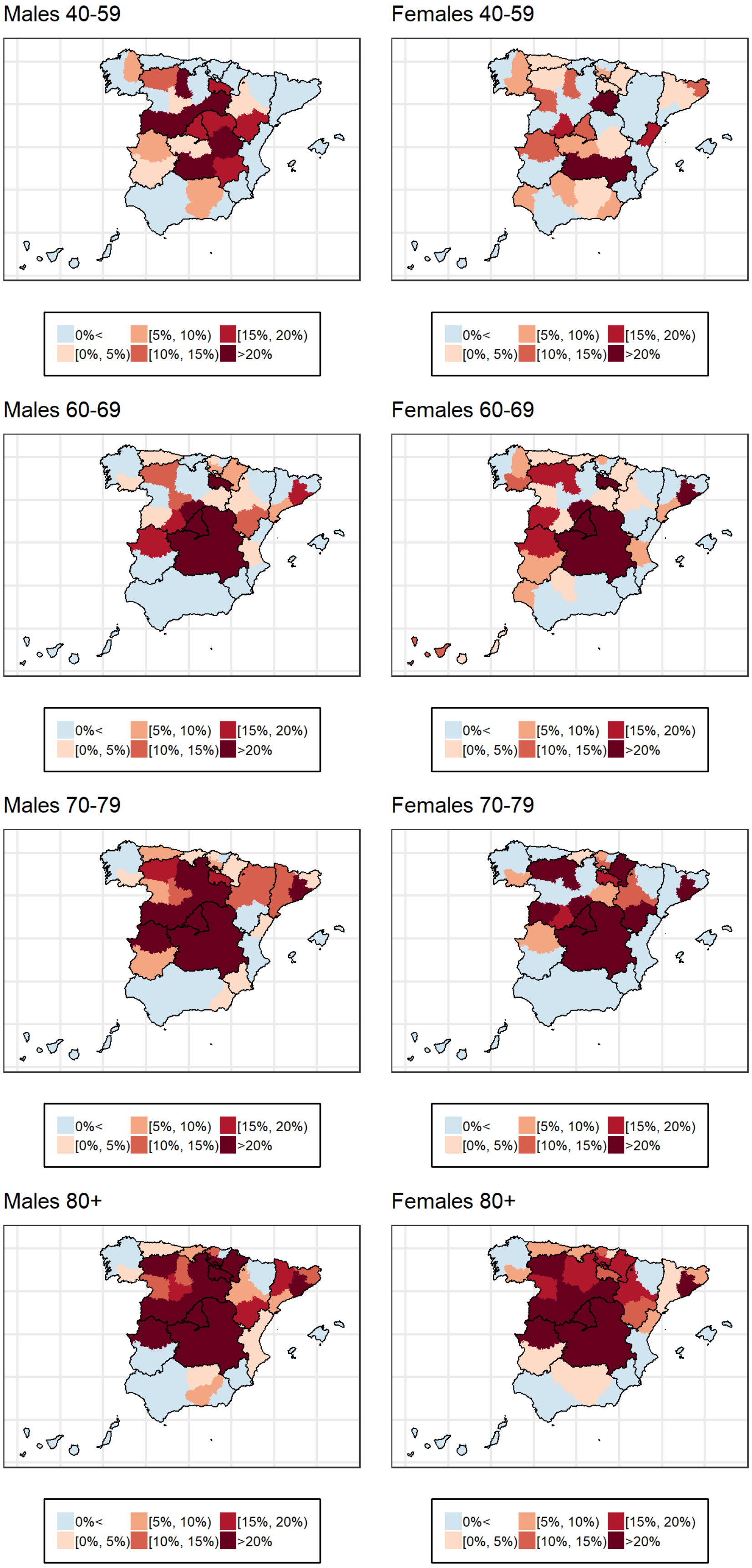

Supplementary Figure 37: Posterior probability that the relative excess mortality is larger than zero during July-December 2020 by NUTS3 regions in Spain stratified by age and sex.

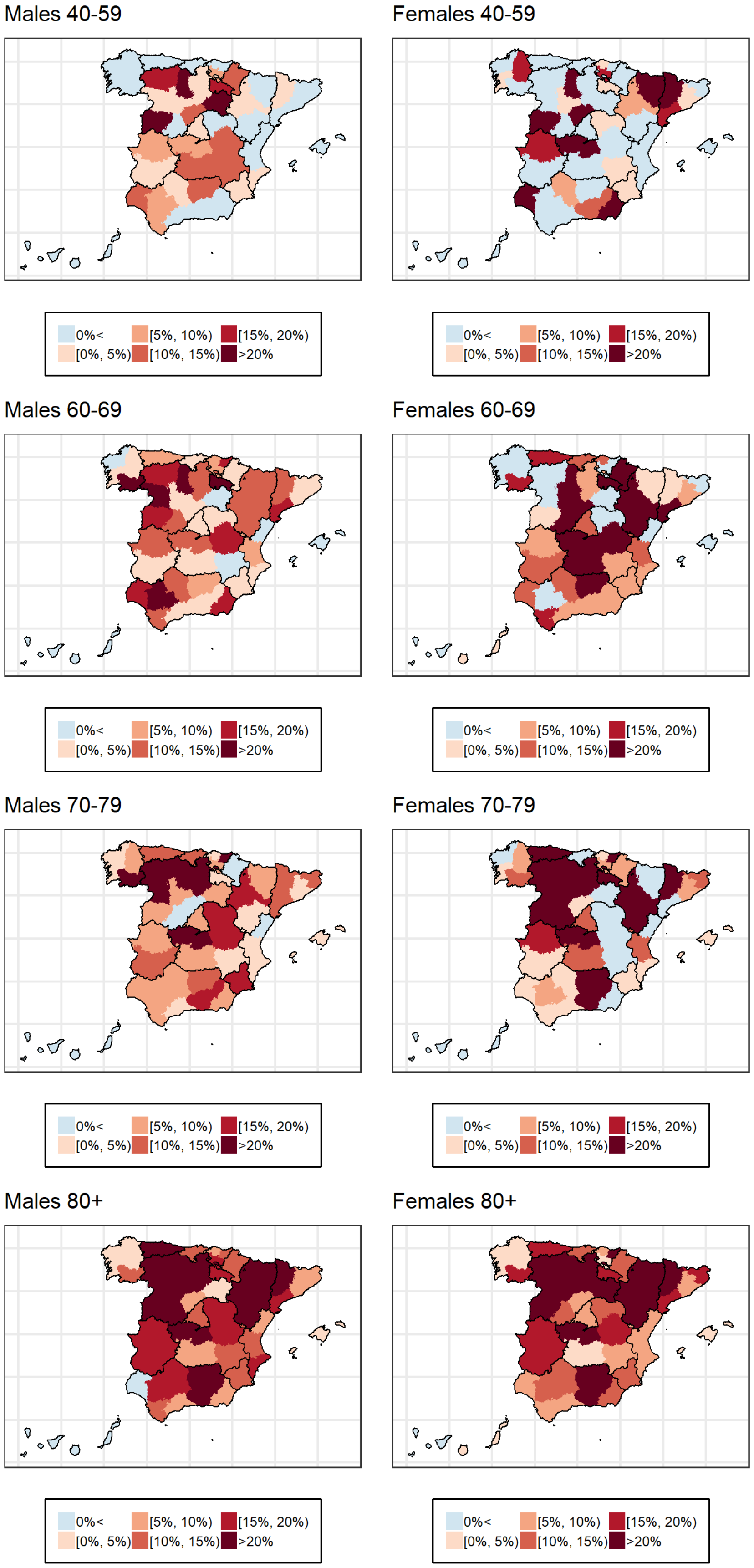

Supplementary Figure 38: Relative excess mortality during January-June 2020 by NUTS3 regions in Switzerland stratified by age and sex.

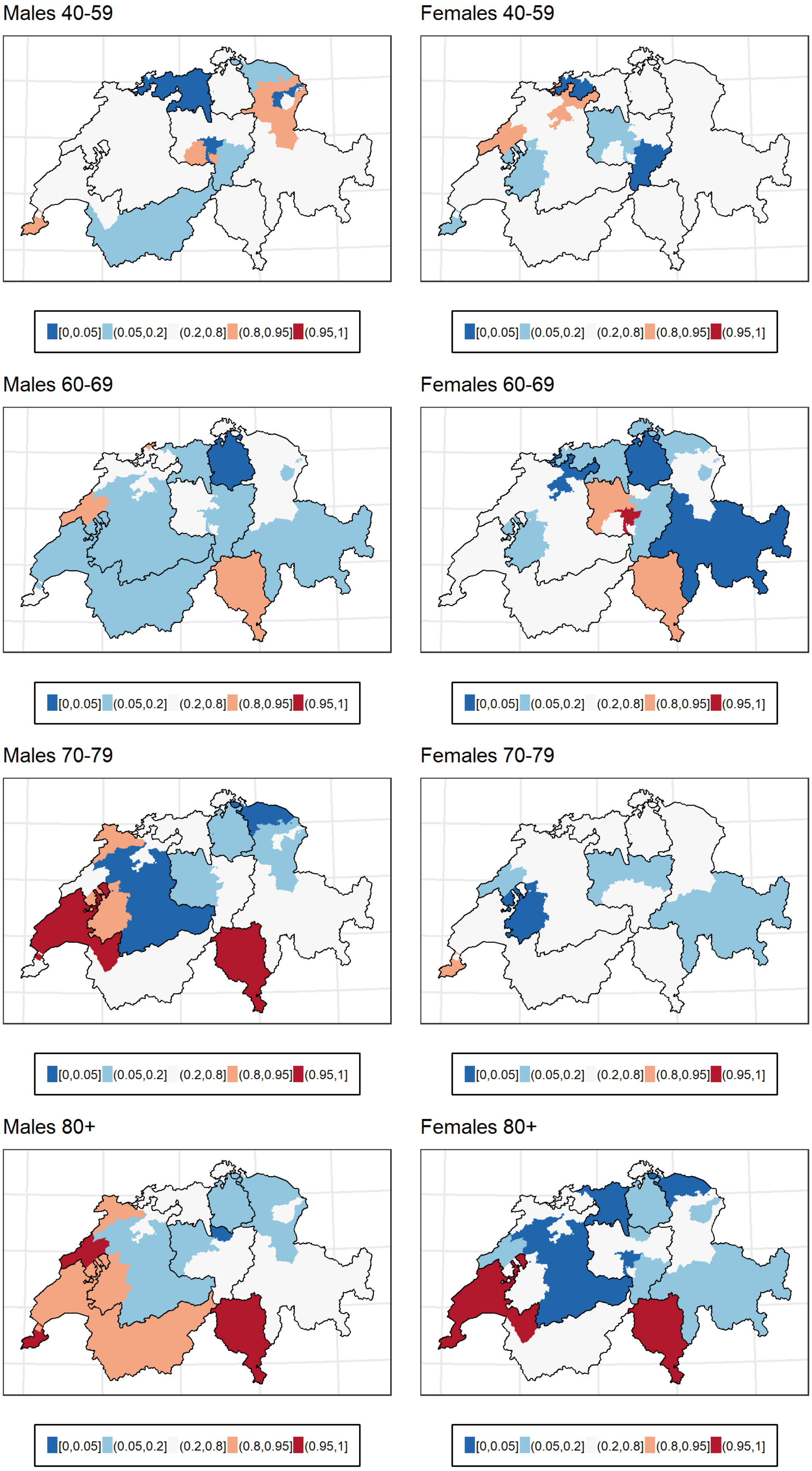

Supplementary Figure 39: Relative excess mortality during July-December 2020 by NUTS3 regions in Switzerland stratified by age and sex.

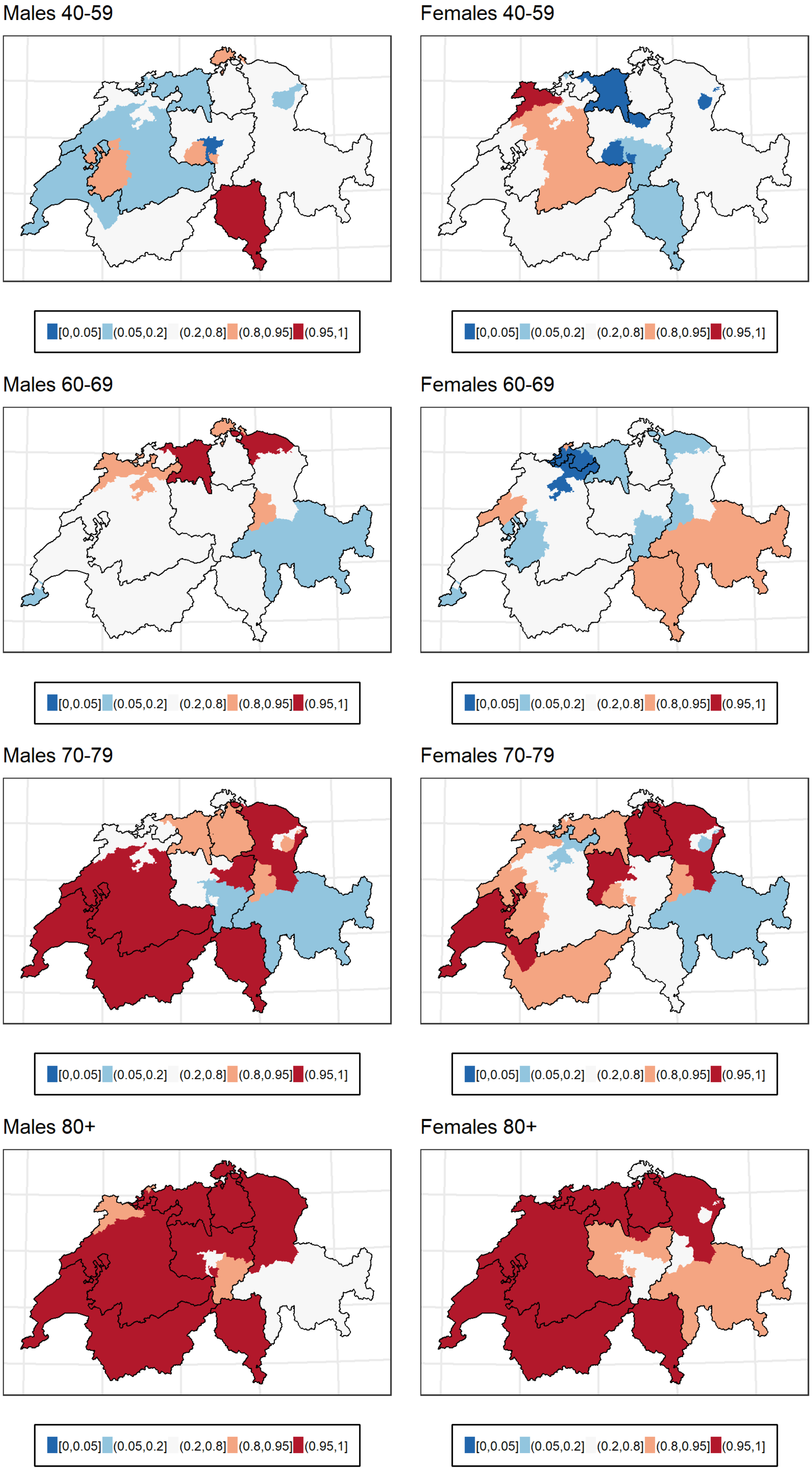

Supplementary Figure 40: Posterior probability that the relative excess mortality is larger than zero during January-June 2020 by NUTS3 regions in Switzerland stratified by age and sex.

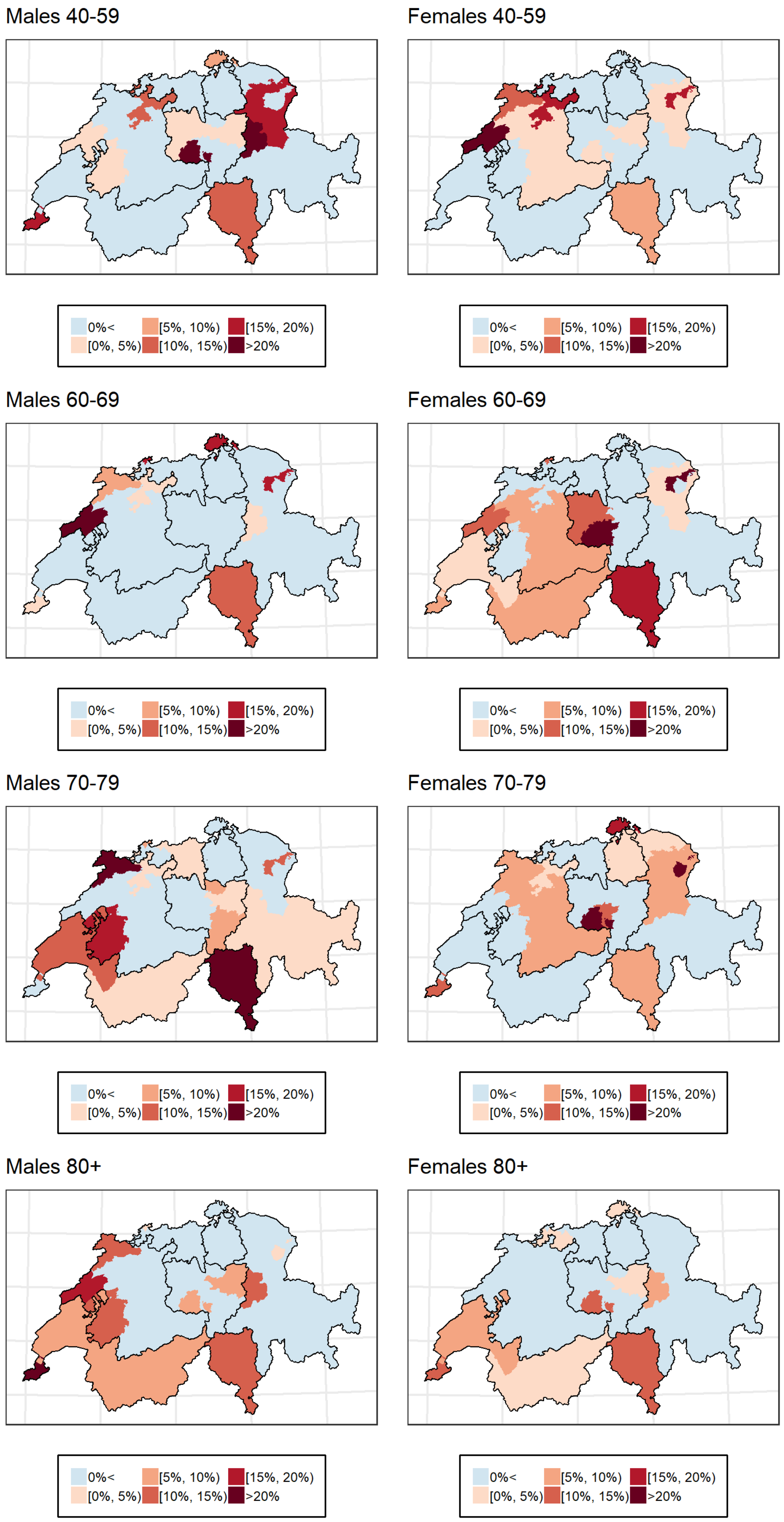

Supplementary Figure 41: Posterior probability that the relative excess mortality is larger than zero during July-December 2020 by NUTS3 regions in Switzerland stratified by age and sex.

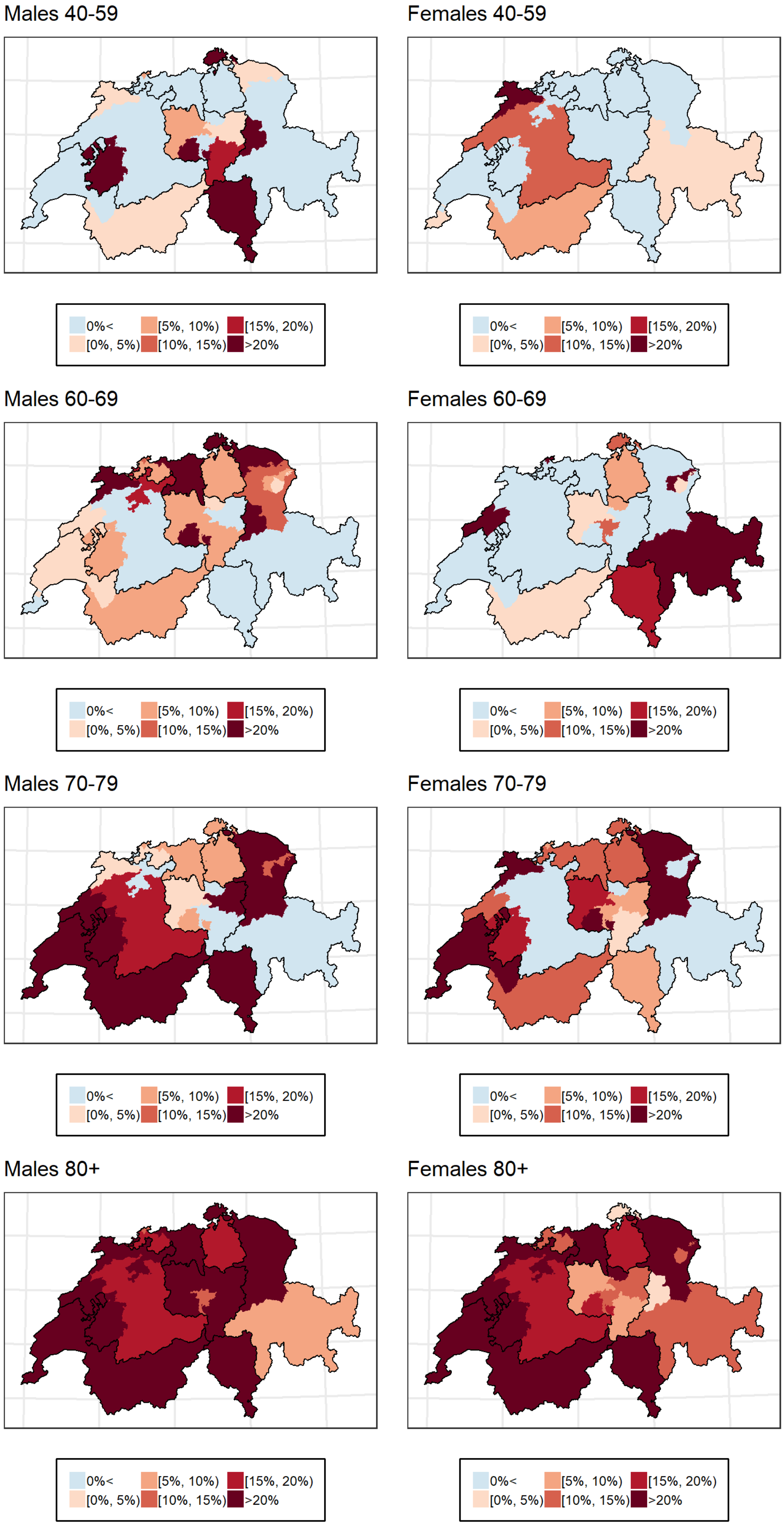

Supplement: Supplementary file 1 — Supplementary Information [file 41467_2022_28157_MOESM1_ESM.pdf]
