# Supplementary material for: Regional excess mortality during the 2020 COVID-19 pandemic in five European countries
Source: Nat Commun. 2022 Jan 25;13:482. doi: 10.1038/s41467-022-28157-3 (PMC8789777; doi:10.1038/s41467-022-28157-3)
Supplement: Supplementary file 3 — Reporting Summary [file 41467_2022_28157_MOESM3_ESM.pdf]

nature portfolio

Corresponding author(s): Garyfallos Konstantinoudis

Last updated by author(s): Dec 8, 2021

Reporting Summary

Nature Portfolio wishes to improve the reproducibility of the work that we publish. This form provides structure for consistency and transparency in reporting. For further information on Nature Portfolio policies, see our Editorial Policies and the Editorial Policy Checklist.

Statistics

For all statistical analyses, confirm that the following items are present in the figure legend, table legend, main text, or Methods section.

n/a	Confirmed	
☐	☒	The exact sample size (*n*) for each experimental group/condition, given as a discrete number and unit of measurement
☒	☐	A statement on whether measurements were taken from distinct samples or whether the same sample was measured repeatedly
☒	☐	The statistical test(s) used AND whether they are one- or two-sided<br>*Only common tests should be described solely by name; describe more complex techniques in the Methods section.*
☐	☒	A description of all covariates tested
☒	☐	A description of any assumptions or corrections, such as tests of normality and adjustment for multiple comparisons
☐	☒	A full description of the statistical parameters including central tendency (e.g. means) or other basic estimates (e.g. regression coefficient) AND variation (e.g. standard deviation) or associated estimates of uncertainty (e.g. confidence intervals)
☒	☐	For null hypothesis testing, the test statistic (e.g. *F*, *t*, *r*) with confidence intervals, effect sizes, degrees of freedom and *P* value noted<br>*Give P values as exact values whenever suitable.*
☐	☒	For Bayesian analysis, information on the choice of priors and Markov chain Monte Carlo settings
☒	☐	For hierarchical and complex designs, identification of the appropriate level for tests and full reporting of outcomes
☒	☐	Estimates of effect sizes (e.g. Cohen's *d*, Pearson's *r*), indicating how they were calculated

Our web collection on statistics for biologists contains articles on many of the points above.

Software and code

Policy information about availability of computer code

Data collection	All data processing and wrangling was performed using the statistical software R.
Data analysis	All data analysis was conducted using the statistical software R (version 4.1.0) and the INLA package (INLA_21.06.11), a package for Bayesian analysis freely available at https://www.r-inla.org/. The code used is also online available at https://github.com/gkonstantinoudis/ExcessDeathsCOVID. We report the results in a form of a shiny web app (https://shiny.rstudio.com/) here: http://atlasmortalidad.uclm.es/excess/.

For manuscripts utilizing custom algorithms or software that are central to the research but not yet described in published literature, software must be made available to editors and reviewers. We strongly encourage code deposition in a community repository (e.g. GitHub). See the Nature Portfolio guidelines for submitting code & software for further information.

Data

Policy information about availability of data

All manuscripts must include a data availability statement. This statement should provide the following information, where applicable:

- Accession codes, unique identifiers, or web links for publicly available datasets
- A description of any restrictions on data availability
- For clinical datasets or third party data, please ensure that the statement adheres to our policy

We provide at https://github.com/gkonstantinoudis/ExcessDeathsCOVID the final version of the datasets for Italy and Switzerland as their mortality data is available online.

Raw mortality data files for Switzerland are provided at https://www.bfs.admin.ch/bfs/en/home/statistics/population/births-deaths/

deaths.assetdetail.19184461.html and https://www.bfs.admin.ch/bfs/en/home/statistics/population/births-deaths/deaths.assetdetail.13187299.html, whereas for Italy at https://www.istat.it/it/archivio/240401. Access to mortality data for Greece, England and Spain is subject to ethics and governance applications. For England, the data were obtained from the Small Area Health Statistics Unit (SAHSU), which does not have permission to supply data to third parties. The data can be requested through the Office for National Statistics (https://www.ons.gov.uk/). For Greece, mortality data can be requested from ELSTAT (https://www.statistics.gr) and for Spain from the National Centre of Epidemiology at the Carlos III Health Institute (https://eng.isciii.es/eng.isciii.es/Paginas/Inicio.html).

Population data is available at the following locations:
England: https://www.ons.gov.uk/peoplepopulationandcommunity/populationandmigration/populationestimates/datasets/populationestimatesforukenglandandwalesscotlandandnorthernireland
Greece: The selected aggregation by age, sex and NUTS3 regions is subject to a request at: https://www.statistics.gr/en/statistical-data-request
Italy: http://demo.istat.it/ricostruzione/download.php?lingua=ita for 2015-2019 and http://demo.istat.it/popres/download.php?anno=2020&lingua=ita for 2020
Spain: https://www.ine.es/jaxiT3/Tabla.htm?t=9691
Switzerland: https://www.pxweb.bfs.admin.ch/pxweb/en/px-x-0102010000_102/-/px-x-0102010000_102.px/

Air temperature at 2m for all countries was retrieved from https://cds.climate.copernicus.eu/cdsapp#!/dataset/reanalysis-era5-land?tab=form.

Field-specific reporting

Please select the one below that is the best fit for your research. If you are not sure, read the appropriate sections before making your selection.

☐ Life sciences ☒ Behavioural & social sciences ☐ Ecological, evolutionary & environmental sciences

For a reference copy of the document with all sections, see nature.com/documents/nr-reporting-summary-flat.pdf

Behavioural & social sciences study design

All studies must disclose on these points even when the disclosure is negative.

Study description	We applied Bayesian spatiotemporal models to historical all cause mortality data (2015-2019) by sex and age group (40<, 40-59, 60-69, 70-79, 80>) to estimate weekly and subnational all cause mortality for the entire 2020, had the COVID-19 pandemic not occurred. We then compared it with the observed mortality in 2020 and retrieved the mortality impact due to the pandemic weekly at the subnational level in 5 European countries.
Research sample	We selected to perform the analysis in European countries for which the impact of COVID-19 pandemic was large (England, Italy and Spain), but also mild (Switzerland) and weak (the first pandemic wave in Greece), which allowed us to examine the effect of the pandemic on total mortality in different contexts. The selection of the countries was also subject to data availability (at the selected aggregation scale, i.e. weekly, NUTS3 regions, age, sex and for the entire 2020) and logistics (such as time to get the data and ethics). We retrieved data for all-cause deaths and population counts from the Office for National Statistics in England, the Hellenic Statistical Authority in Greece, the Italian National Institute of Statistics in Italy, the National Centre of Epidemiology at the Carlos III Health Institute and the Daily Monitoring Mortality System and also the National Statistics Institute and Ministry of Justice in Spain and the Federal Statistical Office in Switzerland. The total number of deaths in 2020 in England was 565,505, in Greece 132,514, in Italy 756,450, in Spain 485,536 and in Switzerland 77,222. The deaths and population under consideration are not a sample but the total number of recorded deaths and population in England, Greece, Italy, Spain and Switzerland during 2015-2020.
Sampling strategy	N/A. We used all reported deaths available for analysis.
Data collection	We obtained all-cause mortality data and population data from the Small Area Statistics Units and the Office of National Statistics in England, the Hellenic Statistical Authority in Greece, the Italian National Institute of Statistics in Italy, the National Centre of Epidemiology at the Carlos III Health Institute and the Spanish Statistical Office in Spain and the Federal Office of Statistics in Switzerland. Temperature data was retrieved from ERA5. Mortality data was available from each country's death certificates, whereas estimation of the general population was performed using mortality, migration, birth data and the latest census. The above statistics are routinely collected, nevertheless the researchers collecting the data were blinded to the study hypothesis. Data harmonization, wrangling and analyses was performed by Garyfallos Konstantinoudis, Michela Cameletti, Marta Blangiardo and Inmaculada León Gómez.
Timing	We used data from 2015 to 2020.
Data exclusions	We did not include European countries for which the current age, sex, weekly and subregional aggregation was not available for analysis or the availability was subject to long waiting times and/or ethics.
Non-participation	N/A. Our data is population based counts of mortality and population based on the national registries and interpolation methods of data from censuses, migration, deaths and birth registries. No participants were involved in the study.
Randomization	Our study is observational and not experimental. We did not use individual participants, rather the complete data on mortality and population in England, Greece, Italy, Spain and Switzerland.

Reporting for specific materials, systems and methods

We require information from authors about some types of materials, experimental systems and methods used in many studies. Here, indicate whether each material, system or method listed is relevant to your study. If you are not sure if a list item applies to your research, read the appropriate section before selecting a response.

Materials & experimental systems

n/a	Involved in the study
☒	☐ Antibodies
☒	☐ Eukaryotic cell lines
☒	☐ Palaeontology and archaeology
☒	☐ Animals and other organisms
☒	☐ Human research participants
☒	☐ Clinical data
☒	☐ Dual use research of concern

Methods

n/a	Involved in the study
☒	☐ ChIP-seq
☒	☐ Flow cytometry
☒	☐ MRI-based neuroimaging
